# Machine learning to estimate the local quality of protein crystal structures

**DOI:** 10.1038/s41598-021-02948-y

**Published:** 2021-12-08

**Authors:** Ikuko Miyaguchi, Miwa Sato, Akiko Kashima, Hiroyuki Nakagawa, Yuichi Kokabu, Biao Ma, Shigeyuki Matsumoto, Atsushi Tokuhisa, Masateru Ohta, Mitsunori Ikeguchi

**Affiliations:** 1 Modality Laboratories Sohyaku Innovative Research Division, Mitsubishi Tanabe Pharma Co., LTD., 1000, Kamoshida-cho, Aoba-ku, Yokohama 227-0033 Japan; 2 Mitsui Knowledge Industry Co., LTD., 2-5-1 Atago, Minato-ku, Tokyo Japan; 3 RIKEN Medical Sciences Innovation Hub Program, 1-7-22, Suehiro-cho, Tsurumi-ku, Yokohama 230-0045 Japan; 4 RIKEN Center for Computational Science, 7-1-26 Minatojima-minami-machi, Chuo-ku, Kobe, Hyogo 650-0047 Japan; 5 Graduate School of Medical Life Science, Yokohama City University, 1-7-29, Suehiro-cho, Tsurumi-ku, Yokohama 230-0045 Japan; 6 Center for Cluster Development and Coordination, Foundation for Biomedical Research and Innovation at Kobe, 1-5-2, Minatojima-minami-machi, Chuo-ku, Kobe, Hyogo 650-0047 Japan

**Keywords:** Computational biology and bioinformatics, Structural biology

## Abstract

Low-resolution electron density maps can pose a major obstacle in the determination and use of protein structures. Herein, we describe a novel method, called quality assessment based on an electron density map (QAEmap), which evaluates local protein structures determined by X-ray crystallography and could be applied to correct structural errors using low-resolution maps. QAEmap uses a three-dimensional deep convolutional neural network with electron density maps and their corresponding coordinates as input and predicts the correlation between the local structure and putative high-resolution experimental electron density map. This correlation could be used as a metric to modify the structure. Further, we propose that this method may be applied to evaluate ligand binding, which can be difficult to determine at low resolution.

## Introduction

Protein structures play an important role in understanding biology. For example, in drug discovery, it is used in structure-based drug design where the binding between a protein and a candidate drug compound is analyzed in detail to improve the compound and deliver a more effective drug^1^.

Many protein structures have been determined by X-ray crystallography or cryogenic electron microscopy (cryo-EM). An electron density map for X-ray crystallography or a Coulomb potential map for cryo-EM is calculated from experimental data, and a protein structure is constructed by placing the atoms of the protein according to the map. In X-ray crystallography, after an initial structure is built, the electron density map is calculated using the structure factors and the protein coordinates. During refinement, the electron density map is updated every time the structure is corrected, and refinement minimizes the residual difference between the calculated and observed scattering intensity data with acceptable geometry. In cryo-EM, the Coulomb potential map is calculated from experimental data but is not updated during refinement. Although the refinement method has not been fully established, it is similar to X-ray crystallography, as it requires a structure that has the appropriate geometry and fits the map well.

**Figure 1:**
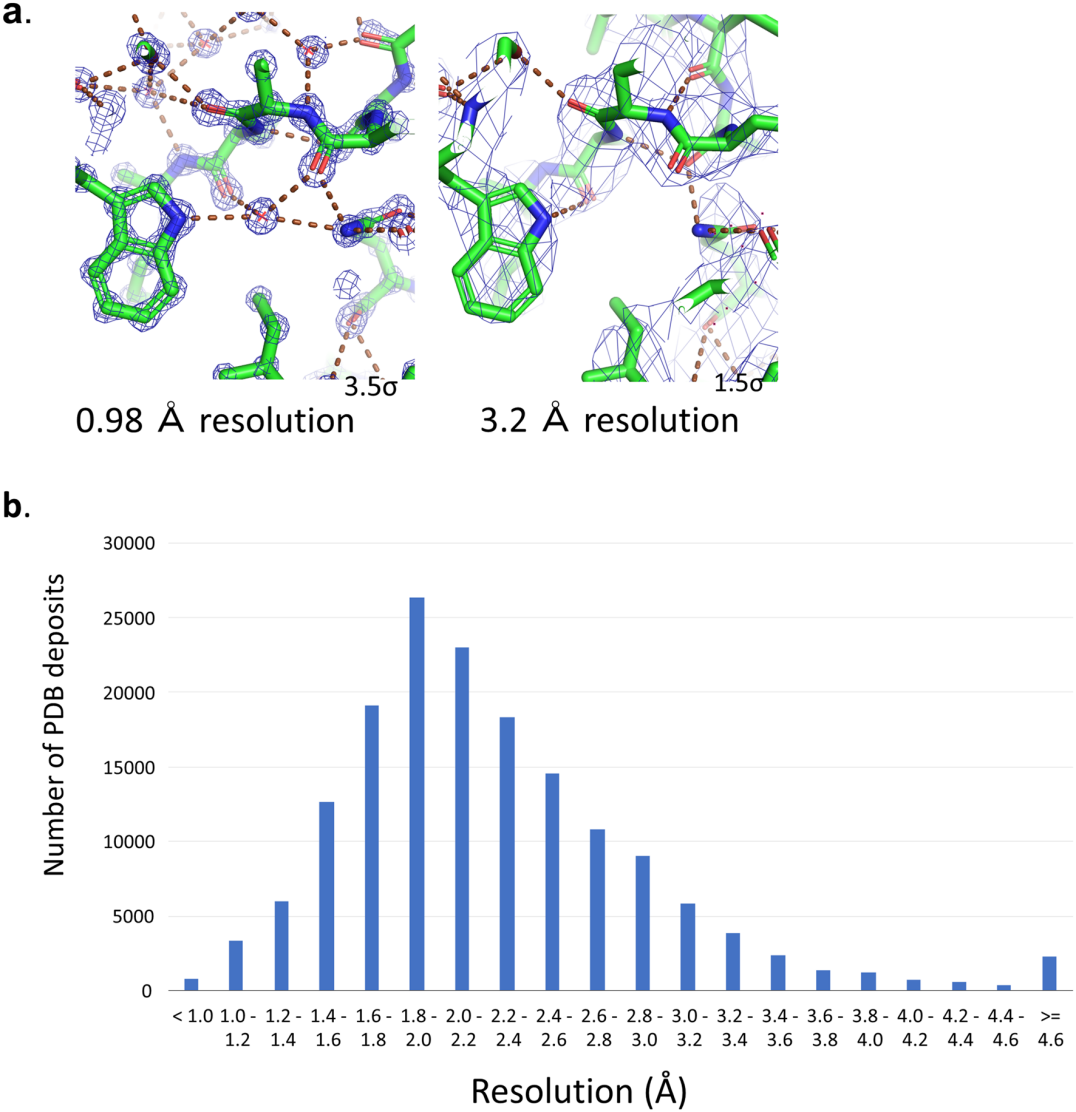
Challenges at low resolution. (**a**) Part of CDK2 structure at high resolution (left; PDB:6Q4G) and low resolution (right; PDB:5UQ1). Atom coordinates and electron density (2mFo-DFc or ρ_obs_) calculated from the coordinates and experimental diffraction data are shown. Although they are almost identical, the hydrogen-bonding network, including water molecules, observed at high resolution is unknown at low resolution. Parts such as side chains that cannot be determined by electron density are often transplanted from high-resolution structure. (**b**) Resolution distribution of structures deposited in the Protein Data Bank (11).

The quality of the electron density and Coulomb potential maps is directly affected by the quality of the experimental data; that is, higher-resolution data result in a clearer map, whereas lower resolution data produce an obscure map (Fig. 1a). In contrast to high-resolution maps, where atomic coordinates can be determined easily, low-resolution maps depend on the experimenter's judgment even when semi-automated assistive tools and prior information are available. However, this may lead to overinterpretation.

In addition, the appearance of maps within a protein molecule can vary. While the secondary structures and the rigid part of the inner molecule provide clear electron density maps, the surrounding or loop regions are often obscured; furthermore, the side chains are often more obscured than the main chain. This is because of the thermal vibration of atoms, multiple conformations, or regional disorder. To construct a structure from maps that are partially obscured, atoms will either be placed or not based on the quality of the electron density map. However, in all these cases, the experimenter needs to make subjective decisions.

Many methods have been proposed to obviate experimenter subjectivity. Refinement software contain a geometry library of amino acids and peptide bonds and perform refinement using the restraints defined in the library^2–4^. In particular, the low-resolution hydrogen bonds in the main chain of a secondary structure can be restrained using additional restraint conditions from a homologous, high-resolution structure in the geometry library^5–7^. However, this method cannot be applied to all cases, because a homologous high-resolution structure is not always available or suitable.

To perform structural checks, methods such as the real-space correlation coefficient (RSCC) for crystal structures^8^ and MolProbity^9^ are available to experimenters. RSCC and MolProbity evaluate the structure on a residue basis. RSCC is a local measure of how well the calculated electron density of an amino acid matches the observed electron density. The RSCC values are large when the electron density map is clear and small for indistinct regions; this correlates well with the temperature factor (that is, B-factor in crystallography), which is an index of the thermal vibration of atoms. After refinement, the atomic position correlates to the electron density, and RSCC would be at its maximum. Thus, RSCC is a measure of structural integrity, rather than a tool for the identification of structural errors and is not used for the correction of crystal structures.

MolProbity, on the other hand, is a software that comprehensively evaluates the geometry of the main and side chains of amino acids and that of their surroundings. It adds hydrogen atoms and optimizes them, and the all-atom contacts and conformations of the main and side chains are evaluated using Ramachandran plot, statistics, and so on.

However, these evaluation methods do not overcome the obstacle of low-resolution electron density map; therefore, if the above-mentioned indicator is judged to be acceptable, no further correction is possible, and the problem of low resolution is not solved. In some extreme low-resolution cases, it may be impossible to determine whether the electron density is attributable to the binding of a compound or noise^10^. For this reason, it is believed that a density map with a resolution of 2.0–2.5 Å or better is necessary for drug discovery or simulation studies^11^. Unfortunately, these criteria are not always met, and only 40% of the registered entities in the Protein Data Bank (PDB) have a resolution better than 2 Å (Fig. 1b)^12^. The accuracy of research based on protein structures would be much improved if the coordinates of the structure could be determined accurately regardless of whether high- or low-resolution data are used.

While the use of cryo-EM structures has been increasingly reported in recent years, its resolution is generally lower than that of crystal structures, limiting its application in drug discovery^13^. Recently, several studies have applied machine learning to the evaluation of protein structures^14^. Examples of the application of a three-dimensional deep convolutional neural network (3D-CNN) to protein structures include the proposal of methods to distinguish the secondary structure in a Coulomb potential map from cryo-EM or to evaluate protein models^15–17^. This shows that 3D-CNN can be applied to protein structures and maps.

In this study, we investigate a novel method, called quality assessment based on an electron density map (QAEmap), that uses machine learning to solve low-resolution problems in structure determination by X-ray crystallography. We decided to use 3D-CNN and train it by using electron density maps and protein structures as input. In our method, the correct structure of a protein is defined as a high-resolution electron density map. We created a new evaluation index called the box correlation coefficient (bCC), which is the correlation between the coordinate structure to be evaluated and the electron density map of the correct structure. QAEmap can predict the bCC with input data of the coordinates and the electron density map used for determining the structure, even when no high-resolution structure is available. This method is also applicable to compound bindings that are unclear. In addition, we compared bCC with RSCC, which is the existing residue-based evaluation method that uses the electron density and coordinates as input.

## Results

### Preparation of protein structures for machine learning

We obtained 9500 sets of protein structure data (coordinate and structure factor files) with a resolution better than 1.5 Å from PDB and set them as candidates of the correct structures for training and test data. Then we selected 22 proteins from them based on conditions, such as whether there are homologous protein structures that could be used as homology modeling templates and whether they belonged to different categories in the CATH classification and so on (see “Methods” and Supplementary Table S1 for more details). Using the coordinates and the structure factor file as a starting point, we created structures containing different errors at various resolutions and the corresponding electron density maps using the crystallographic refinement and homology modeling techniques (see “Methods”). Here, for simplicity, water and other compounds were removed from the initial coordinate files, and only the atoms belonging to the proteins were retained. The electron density maps were 2mFo-DFc electron density maps, which have reduced noise and are commonly used for structure determination in protein crystallography^18^. In addition, a simulated low-resolution correct structure, which was created by refining the original correct structure with truncated structure factors, was added to the protein structure datasets along with the corresponding electron density map.

### Definition of objective variable and box correlation coefficient (bCC)

**Figure 2:**
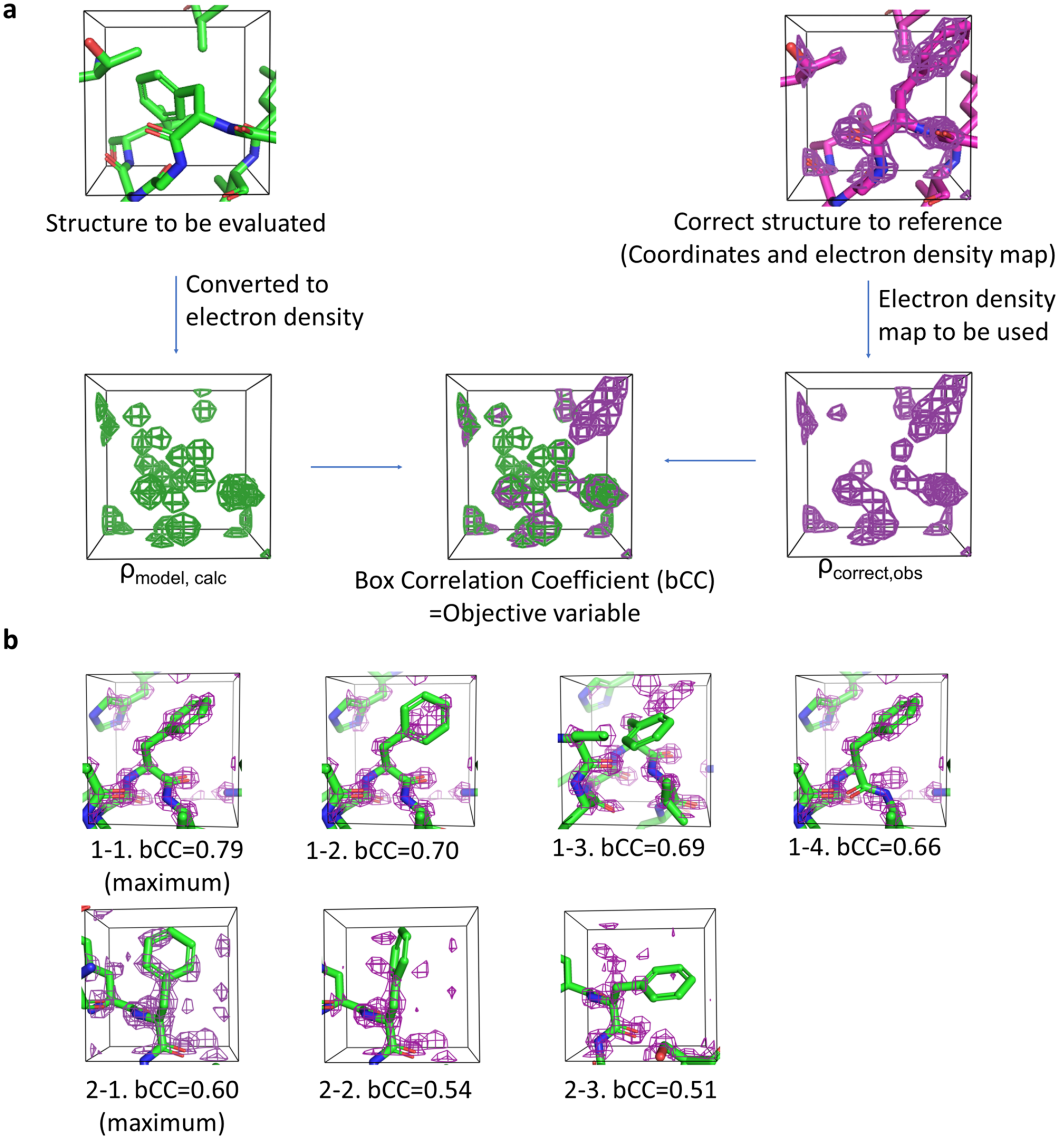
Objective variable. (**a**) Definition of box correlation coefficient (bCC). An example involving a box of size 8.0 Å is shown. The electron density of the correct structure is in magenta, and that calculated from the coordinates to be evaluated is in green. (**b**) Examples of various bCC values, calculated with 1LC0 (resolution, 1.20 Å). Phenylalanine residues were used with maximum bCCs of 0.790 (1-1; Phe256) and 0.595 (2-1; Phe160), the latter was located where the electron density of the correct structure was more blurred than that of the former. The bCC values decreased with fluctuations in the structure (1-2 to 4, and 2-2, 3).

In this study, we extracted amino acids of interest as boxes, used them as training data and calculated an objective variable using the boxes. A metric called bCC was newly defined, and used to evaluate the local protein structure as well as an objective variable in machine learning (Fig. 2a).

Because we defined the high-resolution structure as the correct one, it was necessary to quantify the agreement between the structure to be evaluated and its high-resolution counterpart. Specifically, the electron density map of the correct structure—rather than its coordinates—was used for this quantification. This is because the coordinates of a high-resolution structure are only a model used to describe the electron density map; therefore, the electron density map was considered more appropriate for this purpose.

Hereinafter, this electron density is referred to as ρ_correct,obs_.

The coordinates to be evaluated were converted to electron density using the atom scattering factors in X-ray crystallography to compare with ρ_correct,obs_. The electron density of an atom is distributed depending on the distance from the atom center and a B-factor, according to a Gaussian function^19^.

As we wanted to evaluate the coordinates without considering B-factors, we set the B-factor to be isotropic and fixed at 2.0, which is a sufficiently small value. The electron density of an atom is given by (see “Methods”)

$${\uprho }_{\mathrm{atom},\mathrm{ calc}}\left(\mathrm{r}\right)=\sum_{i=1}^{5}{a}_{i}{\left(\frac{4\pi }{{B}_{iso}+{b}_{i}}\right)}^\frac{3}{2}\mathrm{exp}\left(-\frac{{4\pi }^{2}{r}^{2}}{{B}_{iso}+{b}_{i}}\right),$$

where *r* is the distance from the center of the atom, *a*_*i*_ and *b*_*i*_ are the atomic scattering factors^20^, and *B*_*iso*_ is the isotropic temperature (B-factor) fixed at 2.0.

The electron density ρ_atom,calc_ (*r*) was calculated for all atoms in the structure to produce the ρ_model,calc_ electron density map. Using both the ρ_correct,obs_. and ρ_model,calc_ maps, a cube centered on the center of gravity of the amino acids of interest was extracted. The size and grid of the cubes were arbitrarily determined and were the same for all the residues.

The objective variable is defined as follows with the cubical boxes:

$$\mathrm{bCC}=\frac{\mathrm{ cov }({\uprho }_{\mathrm{correct},\mathrm{obs}}, {\uprho }_{\mathrm{model},\mathrm{ calc}})}{\sqrt{var\left({\uprho }_{\mathrm{correct},\mathrm{obs}}\right)\bullet var({\uprho }_{\mathrm{model},\mathrm{ calc}})}},$$

where *var* is the sample variance and *cov* is the sample covariance.

Because bCC is a correlation coefficient, it ranges from 0 to 1, with values closer to 1 indicating that the electron density and coordinates are well-correlated. The bCC value can be described as follows.

First, bCC would have the maximum value at the location where the coordinates of the correct structure best match the electron density ρ_correct,obs_. However, the location where the maximum bCC is obtained may vary because the thermal vibrations of the protein, multiple conformations, and disorder could affect its location, and electron density noise may also be present (Fig. 2b 1-1, 2-1). Different states of electron density have different possible maxima; therefore, the bCC values have relative implications. The bCC decreases when the evaluated structure deviates further from the correct structure (Fig. 2b 1-2,3,4 and 2-2,3).

### Advantages of bCC

**Figure 3:**
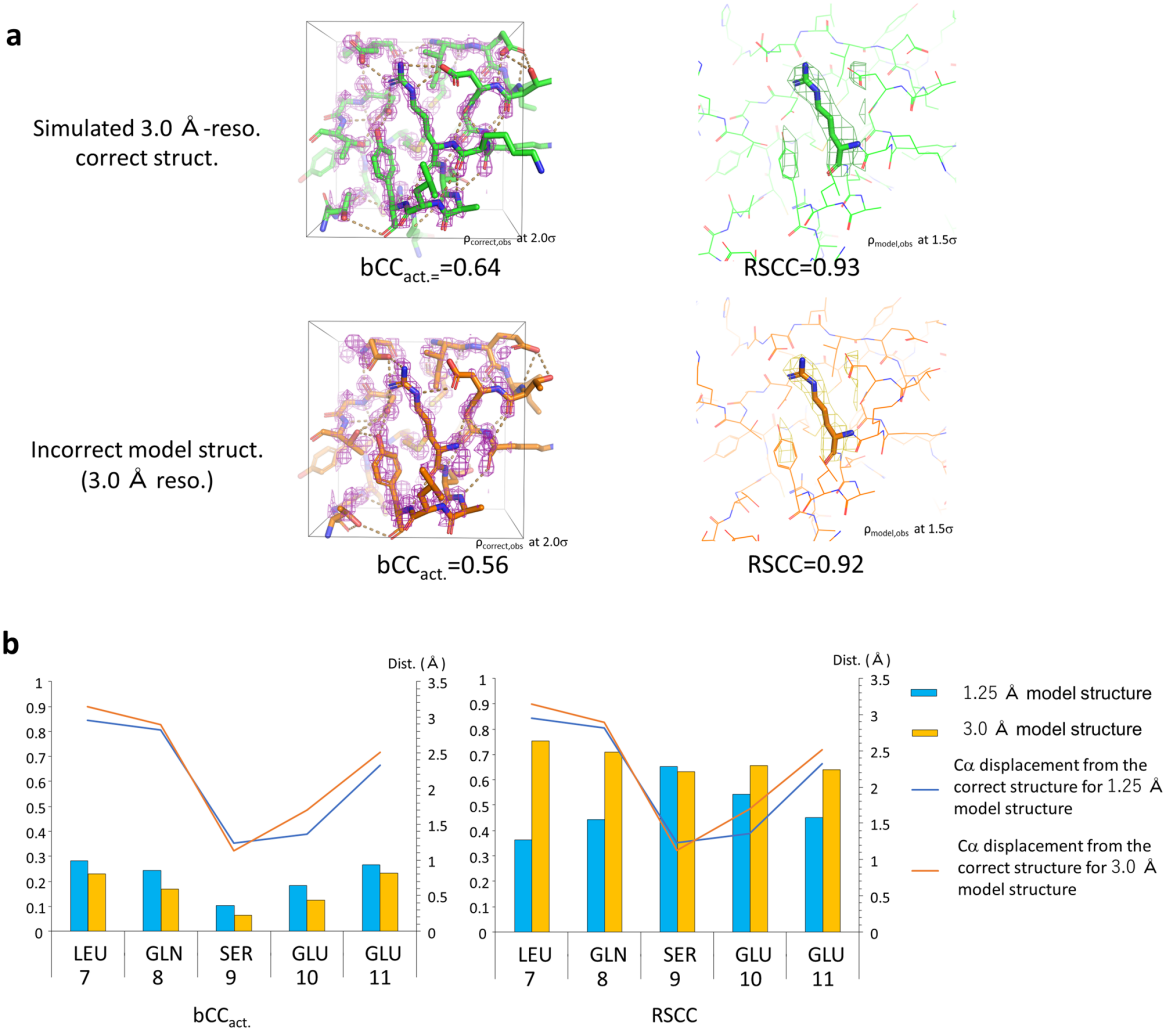
bCC and RSCC. (**a**) Arg67 of 3F9X (molecule D) in the correct and incorrect model structures. The template of homology modeling for the incorrect structure was 1ZKK. Electron densities used for the calculation of bCC (left) and RSCC values (right) are also shown. Values of bCC include the environment surrounding Arg67 (left), while RSCC measured correlation electron density covering Arg67 and its atoms (right). (**b**) Comparison of bCC and RSCC in the region where the main chain is completely misplaced from the correct electron density map. The bCC (left) and RSCC (right) values at residues 7–11 of the model structure at 1.25 Å- and 3.0 Å-resolution structures of 3F9X. The template of homology modeling for the incorrect structure was 5V2N. All the Cα atoms of the region are more than 1 Å away from the corresponding Cα atom of the correct structure. Because bCC is not affected by model bias, no residue is correlated with the correct electron density (bCC < 0.3). Contrastingly, because RSCC is affected by model bias and large B-factors, it shows correlation with electron density, and the effect is larger at 3.0 Å resolution. The electron density maps and coordinates are shown in Supplementary Fig. S1.

There are two advantages of using bCC as an objective variable. First, bCC makes it possible to learn and evaluate amino acids along with their surrounding environment inside the box. It includes non-covalent interactions, such as polar and hydrophobic interactions, with neighboring atoms. In Fig. 3a, the central amino acid fits well to the electron density in both correct and incorrect structures and their RSCCs yield similar high values (~ 0.92). In contrast, the bCC value decreases by ~ 0.1 for the incorrect structure compared with the correct structure because the surrounding environment and interactions with neighboring atoms are markedly different between the two structures. Thus, bCC assesses the coordinate quality of the surrounding atoms and residue of interest. In theory, bCC can include the effects of water molecules in the box. However, because a single oxygen atom contributes less than 1% to bCC in the case of a box of size 12 Å, high prediction accuracy is required for practical use of the estimation of the effects of water molecules.

Second, bCC values are free from model bias and data resolution Model bias, in which electron density maps are affected by the model coordinates because they obtain phase information from them may lead to misinterpretation of the electron density maps. Figure 3b shows the case of incorrect backbones at high (1.25 Å) and low (3.0 Å) resolutions (Supplementary Fig. S1). Despite the large displacements of Cα atoms, RSCCs show that the coordinates correlate with the electron density. This is because of the large B-factors resulting from the misplacement of atoms and model biases that have a significant impact on the low resolution (Fig. 3b (right)). In contrast, bCC is not affected by model biases because it is calculated by referring to the map of the highest-resolution correct structure having the least model bias effect. In fact, bCC exhibits small values at both resolutions, reflecting the large displacements of backbone atoms (Fig. 3b (left)). Therefore, bCC is independent of data resolution and is appropriate as a metric to assess the coordinates.

The box size in this study was 12 Å, which is sufficient to cover the surrounding environment of amino acids. Hereafter, the value of bCC calculated from the actual electron density of the correct structure in the training data is denoted as bCC_act._ and the value of the predicted bCC as bCC_pred_.

### Descriptor preparation and overview of QAEmap

**Figure 4:**
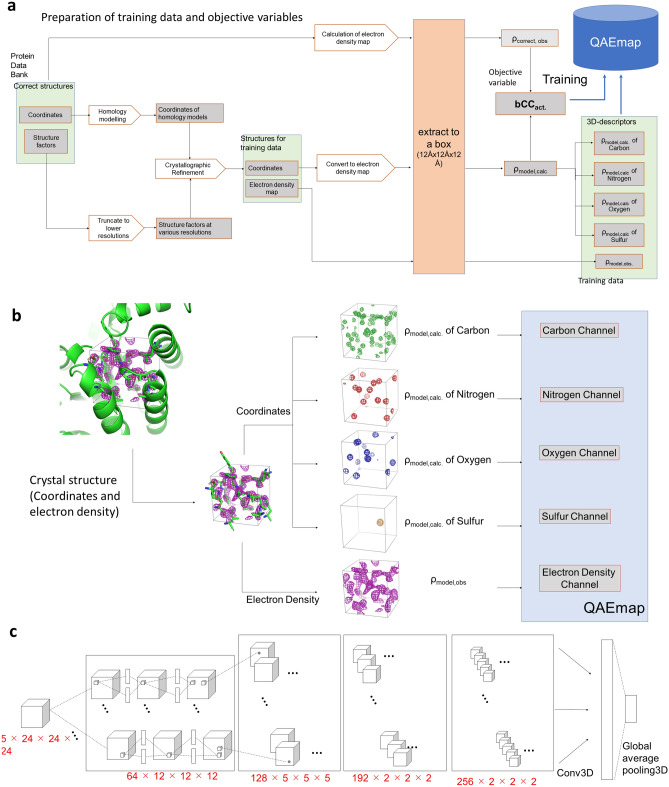
Overview of QAEmap. (**a**) Procedure to generate input data for training. Details are provided in the “Methods” section. (**b**) Examples of three-dimensional descriptors for an amino acid. Five descriptors for each amino acid are prepared as input data. (**c**) Overview of QAEmap architecture for learning one amino acid type. It consists of four convolutional neural network (CNN) layers followed by the maximum pooling or average, and the dropout that was inserted before the last convolution layer. Twenty identical learning models were prepared for twenty amino acid species.

We created three-dimensional descriptors from the coordinates and electron density map of the prepared structures described above. Both these descriptors were cut into 12 Å cubes for all the amino acids in the structure (Fig. 4a, b). The coordinates were further divided by atom species and converted into electron density using the objective variable calculation. These were then used as the input data for training as three-dimensional descriptors. As the created structures corresponded to the correct structures in the initial state, bCC_act.,_ defined earlier, could be calculated.

The amount of input data for each species differed depending on the amino acid, but no adjustments were made, and the data for all 263,689 amino acid residues were used (Supplementary Fig. S2).

Our QAEmap 3D-CNN architecture was based on SqueezeNet^21^ and was implemented using TensorFlow (v. 1.6.0) (Fig. 4c). Twenty models with identical architecture were prepared and trained for 20 amino acid species. These models were trained separately and independently to learn the structures and surrounding environmental characteristics of each amino acid. All training procedures and parameters were the same for all amino acid types, the initial hyperparameter values were used, and the learning rate was set to 1e−05. The three-dimensional input data were rotated to all possible orientations at intervals of 90°, i.e., the model was trained with the objective variable for all 24 rotations. The QAEmap model was trained until convergence, which was reached after 40 epochs.

### Evaluation of QAEmap on test data

**Figure 5:**
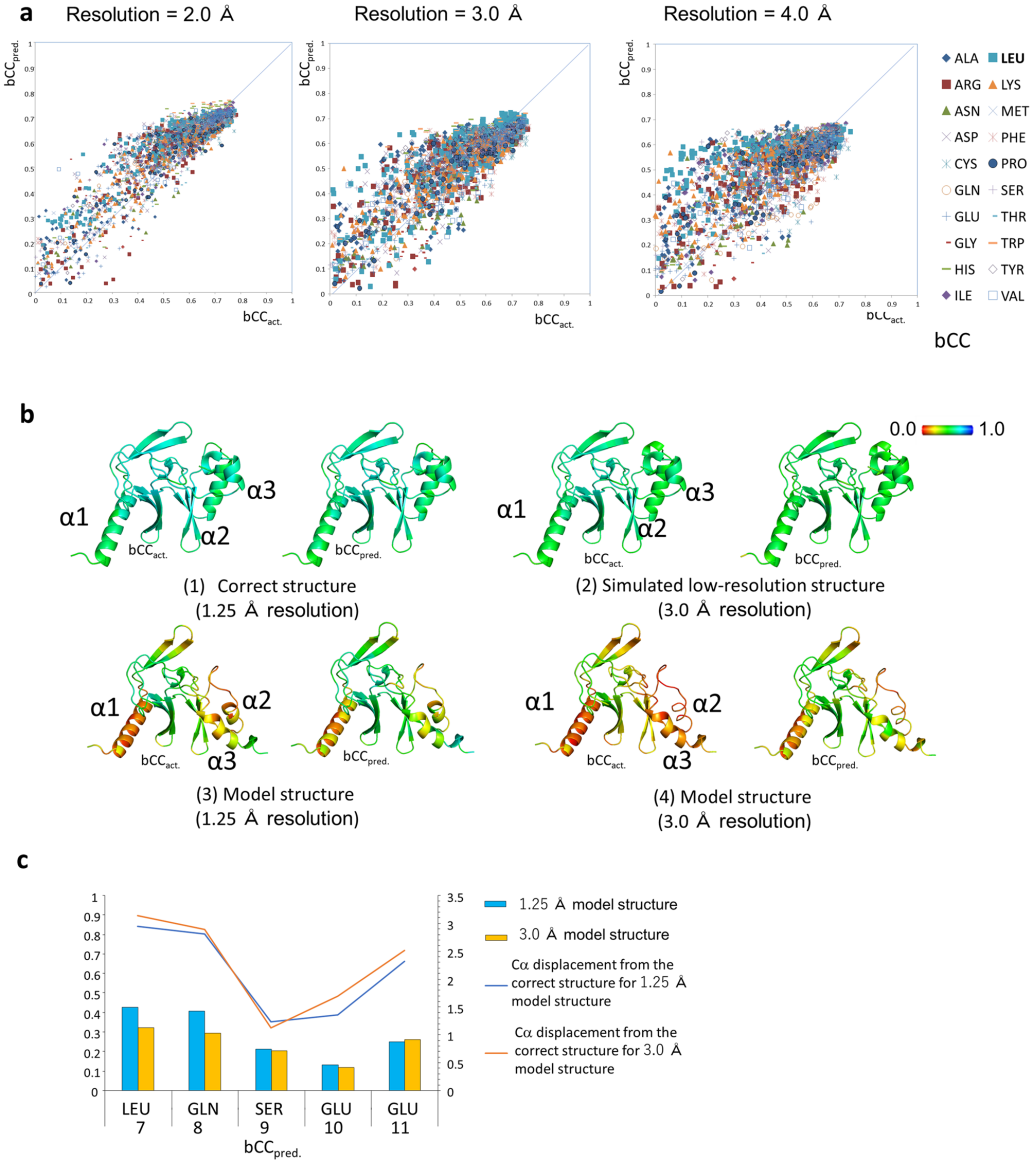
Evaluation of QAEmap with test data. (**a**) Correlation between bCC_act._ and bCC_pred_. The correlation coefficients are 0.890 at 2.0 Å, 0.848 at 3.9 Å, and 0.736 at 4.0 Å resolution for all amino acids. (**b**) bCC_act._ and bCC_pred._ for the correct structure of PDB: 3F9X (1), Simulated 3.0 Å-resolution structure (2); model structures at 1.25 Å (3) and 3.0 Å (4) resolution. The structures are presented with bCC values in rainbow color. (**c**) The bCC_pred_. values of the model structures of Fig. 3b. Although the coordinates and electron density of the input are the same as those of the RSCC (Supplementary Fig. S1 (right)), the bCC_pred._ values show no correlation with the correct electron density with bCC_pred._ values <  ~ 0.4.

We performed prediction calculation of the test data consisting of six proteins and compared the predicted values with the actual values (Supplementary Table S1 and Fig. 2). The correlations between bCC_pred._ and bCC_act._ were observed per amino acid for three different resolutions (Fig. 5a, Supplementary Figs. S3 and S4). The correlation coefficients varied among the amino acids, and the maximum and minimum observed correlation coefficients were 0.886 for Valine and 0.755 for Histidine, respectively. The correlation decreased for amino acids with lower resolutions; for amino acids with resolutions at 5.0 Å—such as cysteine, histidine, glutamine, lysine, and methionine—it decreased to 0.6 or even lower values.

Subsequently, we compared the correct structure of 3F9X and its simulated low-resolution structure at 3.0 Å resolution (Fig. 5b and Supplementary Fig. S5). The two structures were almost identical with a root-mean-square difference (RMSD) of 0.16 Å for all atoms, and the bCC_act._ values exceeded 0.6 for almost all amino acid residues. When bCCs were predicted using the electron density ρ_model,obs_ of each resolution as input data, bCC_pred._ correlated well with bCC_act._ (for the correct structure, the average difference between bCC_pred._ and bCC_act._ was − 0.016 and standard deviation was 0.020, and for the simulated low-resolution structure, the average difference and standard deviation were 0.041 and 0.029, respectively). It was shown that the bCC of the correct coordinate structures could be estimated independent of the resolution.

As examples of incorrect structures, we examined two model structures, which were refined 5V2N-templated homology models, against the structure factors for 1.25 Å and 3.0 Å resolution. With the exception of the two terminal residues, no residue exceeded the correct structure’s bCC_pred._, and the structure with the maximum bCC_pred._ was the correct structure, as expected.

The model structures had conformational errors at α1–3, and the bCC_act_. values were as low as 0.2–0.4 (Fig. 5b and Supplementary Fig. S5). Most of the bCC_pred_. values were 0.4 or less, implying that they were precisely predicted and not correlated with the correct electron density. The bCC values of the region of Fig. 3b were also predicted, and no correlation with the high-resolution electron density was predicted (bCC_preds._ <  ~ 0.4) correctly (Fig. 5c). However, the bCC_pred._ value of some residues was as high as 0.6, which is approximately 0.2-fold higher than that of bCC_act._, and these structures were predicted to be well-correlated with the correct structure, despite the main chain being incorrect [e.g., for the 1.25 Å resolution structure of Ile154, bCC_act._ = 0.395 and bCC_pred._ = 0.668, and for the 3.0 Å resolution structure of Lys150, bCC_act._ = 0.315 and bCC_pred._ = 0.583 (Supplementary Fig. S5)]. This problem should be considered and solved as follows.

When predicting bCC for the correct structure in cases where the main chain is incorrect, it is sufficient to indicate that no correlation exists. For residues where the main chain is almost correct or well-correlated to the correct structure, it is necessary to predict the relative bCCs precisely, that is, to distinguish which state is better correlated to the correct structure. In future work, we will proceed to optimize the training data and training of QAEmap further for identifying residues that can be corrected with bCC_pred._ and improving the prediction accuracy of bCC within the structural correction range.

The other model structures of the test data were evaluated by QAEmap; the results are shown in Supplementary Fig. S6.

### Evaluation of QAEmap with actual experimental data

An actual low-resolution structure was evaluated using QAEmap. Because test data were obtained by truncating high-resolution data, the signal-to-noise ratio of actual structure factor data would be worse than that of the test data at the same resolution.

The CDK2: Spy1 complex (3.2 Å resolution, 288 residues;^22^), registered in PDB as 5UQ1, was evaluated, and 2R3F (1.5 Å resolution;^23^) was used for comparison as a high-resolution structure of CDK2. They were determined using the molecular replacement method with a homologous CDK2 structure as a template and differ in crystal form and conformation; the RMSDs between all corresponding atoms were 3.01 Å.

**Figure 6:**
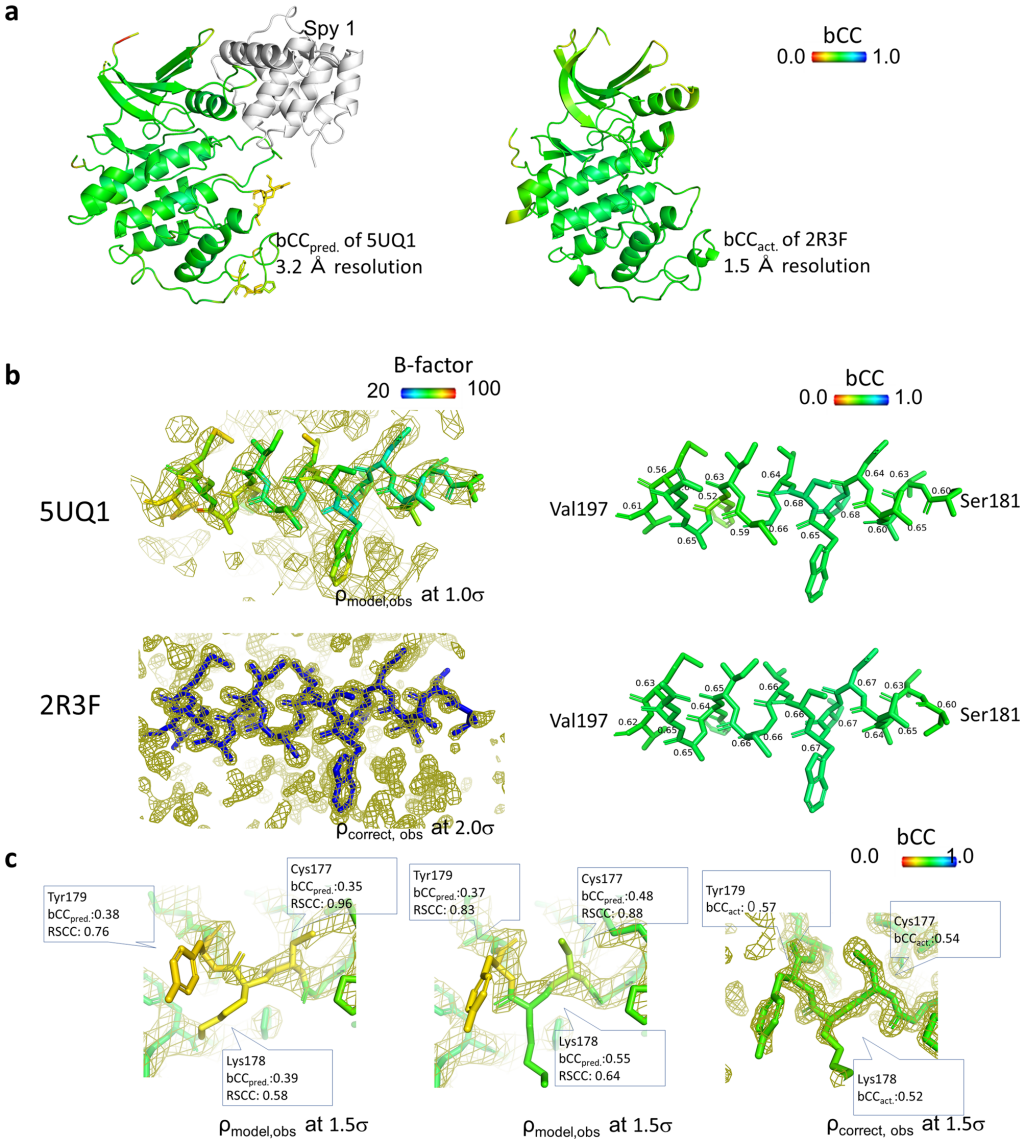
Comparison between high- and low-resolution structures of CDK2. (**a**) The whole structures 5UQ1 (3.2 Å resolution; complexed with Spy1) with bCC_pred._ and 2R3F (1.5 Å resolution) with bCC_act._ in rainbow color. (**b**) The same a helix from Ser181 to Val195 in 5UQ1 (above) and 2R3F (below). Despite the electron density and B-factors varying between the two structures because of the resolution (left), bCC_pred._ for 5UQ1 and bCC_act._ for 2R3F are consistent (right; bCC values are shown). It means the accuracy of the coordinates can be predicted independent of resolution. (**c**) 5UQ1’s bCC_pred._ is lower than 2R3F’s bCC_act._ from Cys177 to Tyr179. The electron density is unclear because of the loop region, and the direction of the carbonyl at Lys178 cannot be determined based on the electron density. Because bCC_pred._ of Lys178 is low at 0.39, it was modified in the same direction as 2R3F, which improved bCC_pred._, predicting that the modification was correct.

The bCC_pred._ of 5UQ1 was predicted by QAEmap, and the bCC_act._ of 2R3F was calculated; the mean bCC values were 0.592 and 0.593, respectively (Fig. 6a, Supplementary Fig. S7). The individual bCC_pred._ values were in good agreement with the bCC_act._ of 2R3F (Fig. 6b). Specifically, the secondary structures of the C-terminal domain and QAEmap predicted that the structure of 5UQ1 is as accurate as that of 2R3F.

When the bCC values were compared locally, the region of amino acid residues 177–179 had bCC_act._ values of 0.52–0.57 for 2R3F and 0.35–0.39 for 5UQ1. Structural modification mimicking 2R3F and using bCC_pred._ values as an index improved the bCC_pred._ of Lys178 and resulted in a more accurate prediction (Fig. 6c).

In addition, the bCC values were higher when packed with neighboring molecules than when exposed to solvents (Supplementary Fig. S8), indicating that local differences in structures at different resolutions can be described using bCC.

### Application of QAEmap to compound-bound structures

Another challenge at low resolution is determining ligand binding and the binding mode. QAEmap can be applied in these instances to evaluate compound binding, as an input box contains the environment around the amino acid of interest. We attempted to assess the binding of compounds using our trained QAEmap. Although atoms belonging to compounds were removed from the current training data, ligand compounds consisting of carbon, oxygen, nitrogen, and sulfur could be treated as part of the surrounding environment because the channels of QAEmap were designed for these four atom types.

**Figure 7:**
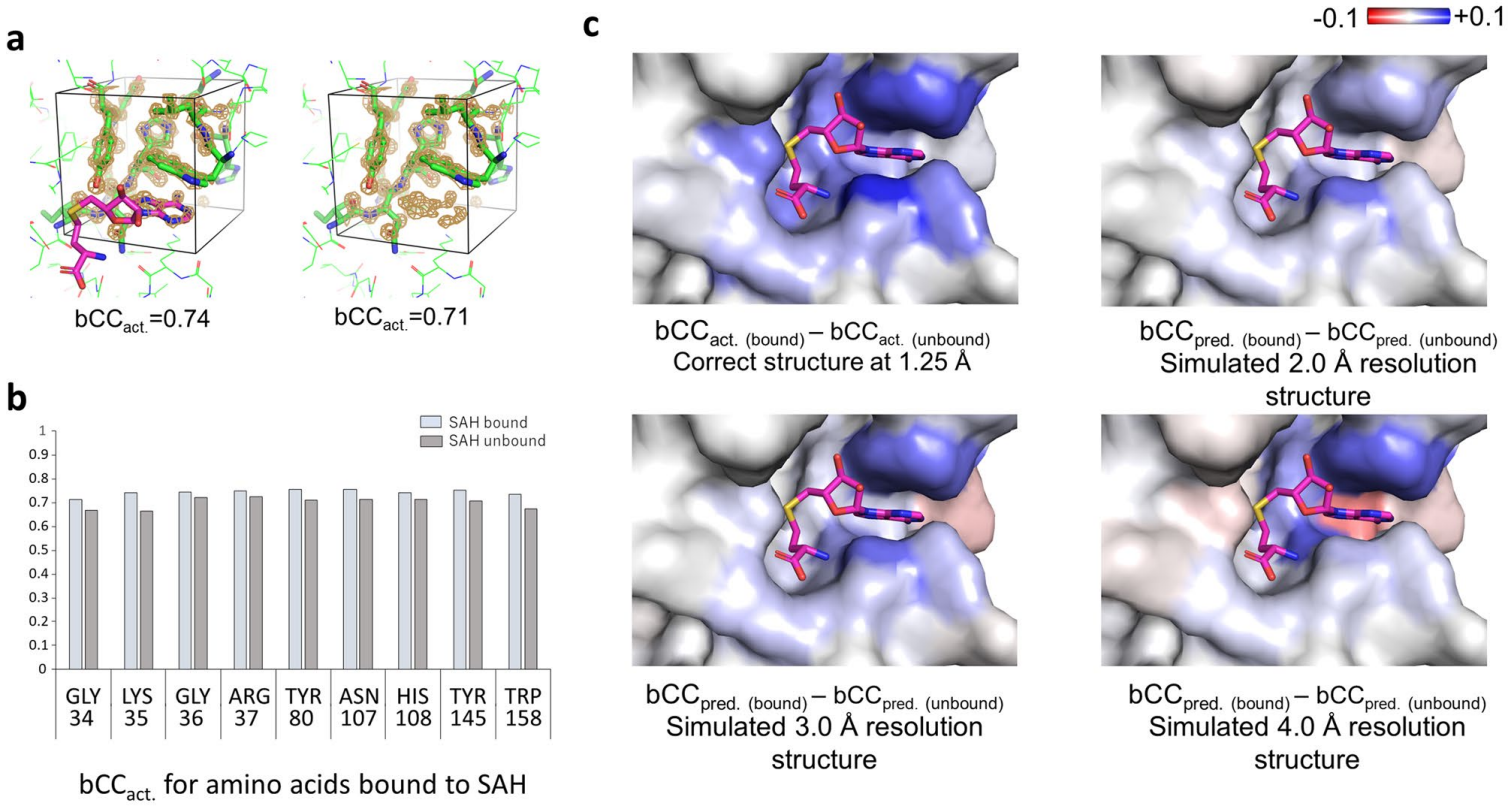
Application of QAEmap for a compound binding. (**a**) Boxes with (left) and without (right) SAH of His108 of SET domain protein methyltransferase (PDB: 3F9X, at 1.25 Å resolution) neighboring a SAH molecule (magenta), which is an input for QAEmap, includes SAH partially. There are a total of nine residues neighboring SAH. (**b**) Values of bCC_act._ for the nine residues with and without SAH. (**c**) Surface presentation colored by the differences between SAH-bound and SAH-unbound of bCC_act._ of the correct structure, or bCC_pred._ of simulated low-resolution structures at resolutions in the range of 2–4 Å. The scores of bCC indicate the binding structure is preferred.

Figure 7a shows an example of the SET domain protein methyltransferase (PDB: 3F9X) bound with *S*-adenosylhomocysteine (SAH). For amino acids adjacent to SAH, the bCC_act._ values were approximately 3–8% higher in the model with SAH than in the model without SAH (Fig. 7b). If these differences can be predicted by QAEmap, then the binding and docking pose can also be predicted.

To test this hypothesis, we prepared bound/unbound structures, refined them to make simulated low-resolution structures at 2.0, 3.0, and 4.0 Å resolutions, and predicted bCC_pred._ using QAEmap. As the electron density of a compound depends on the existence of a compound in the structure, the determination of the presence or absence of the compound from the electron density is arbitary, especially at resolutions of 3.0 Å and 4.0 Å (Fig. 7c and Supplementary Fig. S9).

On the other hand, when the difference between bound and unbound bCC_pred._ was calculated, the bound structure was predicted more accurately at all resolutions. This suggests that QAEmap could be used to determine the binding of compounds.

As bCC_pred._ reflects the docking pose of the compound, an accurate docking pose is required for the determination. In addition, as some atom types and interactions between a compound and a protein are specific, it is necessary to prepare training data that can be used to train QAEmap on the compound binding states for QAEmap to be applied to compound binding.

## Discussion

We developed a method to evaluate the local structure of protein crystals independent of the resolution of the experimental data. Further development is ongoing to expand the resolution of prediction, assign water and other compounds, and improve prediction accuracy.

We currently intend to devise a method to improve coordinates based on the predicted bCC. By modifying the coordinate structure to improve the predicted bCC, the electron density calculated from the coordinates is expected to match the high-resolution map more closely.

We are also considering the application of QAEmap to cryo-EM. Cryo-EM has fewer entries in PDB than X-ray crystallography, and few have a resolution above 1.5 Å^12^. As the data and their tendency for cryo-EM differ from those for X-ray crystallography, it is necessary to adjust QAEmap for cryo-EM data. However, the basic approach of using maps and coordinates as inputs and bCC as an objective variable is still applicable.

QAEmap is an integrated local structure assessment tool that can be used by structural protein experimenters to confirm structural determination. Further, it can be used by structure users to guide the viewing of the structure and can become a useful tool for the expanding structural biology community.

## Methods

### Preparation of training data

Approximately 9500 entries with resolutions better than 1.5 Å, containing more than 30 amino acids and consisting of only l-amino acids were extracted from the PDB. For entries from identical proteins, one was selected, and for multiple chains included in an entry file, only one of the chains was selected. Next, we selected entries whose structure factor file and crystallographic information were available and excluded entries containing nucleic acids. Then, we searched for their homologous protein structures in the PDB, which could be used as templates for homology modeling with BLAST search bit score > 100^25^ and RMSD values < 1 Å between the Ca atoms of the homologous and target proteins. We obtained 511 target proteins, which we classified using CATH^24^. Further, 67 proteins were evenly selected from the C (Classification) category levels 1–3, avoiding the same A (Architecture), T (Topology) and H (Homologous superfamily) levels and including more Cys, Trp and Met residues that are relatively rare to the other amino acids in most proteins. Homology models were built using MODELLER^26^ with the homologous proteins having more than 40% sequence identity as templates. The models superimposed on the target structure in the crystal coordinate system were the target model structures. They were refined against the structure factors for resolutions ranging from the highest to 5.0 Å in increments of 0.5 Å by using the DIMPLE in the CCP4 package^27^ over 100 cycles with all the default restraint conditions. For each initial model, 8–9 model structures were prepared. Their corresponding electron density (ρ_model,obs_) maps were also calculated from the refined coordinates and the structure file. Test data were prepared with the same method from 6 proteins (Supplementary Table S1).

### Preparation of three-dimensional descriptors

The B-factors in the coordinate files were set to 2.0 using PDBSET (CCP4), and the coordinates were divided based on atom types. The coordinate files of each atom type were then converted into electron density maps using the ATOMMAP mode of SFALL (CCP4) and extended to the unit cell using MAPMASK (CCP4). All the electron density maps, ρ_model,obs_ and ρ_model,calc_, of each atom type were cut into cubic boxes with sides of 12 Å and a grid size of 0.5 Å by MAPROT (CCP4); they were centered on the center of gravity of an amino acid. There were 315,187 amino acids in total for the training data. Thus, five descriptors for each amino acid were calculated and assigned to the different channels in the QAEmap model.

### Calculation of objective variables

The coordinates of all the atom types in the model structures were converted into electron density in the same manner as mentioned above. The electron density (ρ_model,calc_) and electron density of the correct structure (ρ_correct,obs_) were cut into boxes, as with the three-dimensional descriptors. The correlation coefficient between the boxes over all grids was calculated as bCC_act_.

### Determination of training data

We prepared training datasets with 20–30 target proteins from the 67 proteins described above, having 10,000 or more data units per amino acid. We trained the learning models described in the text with the training datasets and predicted the objective variables of the test data from 1LC0 and 3F9X. We recognized that an unbiased distribution of object variables in the training data is important for performance. Therefore, we examined the training datasets in terms of the unbiased distribution of the object variables.

We ultimately selected the proteins and their corresponding homologous proteins presented in Supplementary Table S1 for the training data. For the 22 target proteins, 96 homologous proteins were used as templates, and 1023 model structures and a total of more than 260,000 training data units were created by the method described in the text. Then we prepared five descriptors for each amino acid of the training data resulting in a total data volume of 74 GB.

### Data preparation for CDK2

5UQ1 (3.2 Å resolution) and 2R3F (1.5 Å resolution) were downloaded from the PDB along with their structure factor files. All water and ligand molecules were removed from the coordinate files. Ten refinement cycles were performed by REFMAC5 (CCP4)^28^. The R-factor and free R-factor were 0.192 and 0.277 for 5UQ1, and 0.262 and 0.292 for 2R3F, respectively. The values of bCC_act._ were calculated with the data of 2R3F as the correct structure. After the structural modification of 5UQ1, bCC was refined and predicted in the same manner. The modified structure’s R-factor was 0.190, and its free R-factor was 0.276.

### Calculation of RSCC

The RSCC of the test data of 3F9X and 5UQ1 was calculated using EDSTATS (CCP4), the ρ_model,obs_ electron density map, and the coordinates after refinement.

### Data preparation for SAH-bound and -unbound 3F9X structures

3F9X (1.25 Å resolution) was downloaded from the PDB, along with its structure factor file. All water molecules were removed from the coordinate files, following which two coordinate files were prepared; one was with an SAH molecule, and the other was without. They were refined against 1.25 Å, 2.0 Å, 3.0 Å, and 4.0 Å-resolution structure factors by REFMAC5 over 10 cycles. The R-factors and free R-factors of the SAH bound structures were 0.276 and 0.293 for 1.25 Å, 0.271 and 0.288 for 2.0 Å, 0.260 and 0.269 for 3.0 Å, and 0.252 and 0.261 for 4.0 Å resolution, respectively. For the SAH unbound structures, the R-factors and free R-factors were 0.278 and 0.300 for 1.25 Å, 0.273 and 0.291 for 2.0 Å, 0.262 and 0.269 for 3.0 Å, 0.256 and 0.259 for 4.0 Å resolution, respectively. The values of bCC_act._ were calculated with the data of the 1.25 Å-resolution structures.

## Software

The software package PyMOL (The PyMOL Molecular Graphics System, v. 2.3, Schrödinger, LLC, https://www.pymol.org/2/) was used for the visualization of protein structures and maps; MODELLER (v. 10.1, University of California San Francisco, https://salilab.org/modeller/) and CCP4 (v. 7.0, Collaborative Computational Project No. 4, https://www.ccp4.ac.uk/) were used for creating protein structures and map files. TensorFlow (v. 1.x, Google Brain, https://www.tensorflow.org/install/pip) and Python (v. 3.6, Python Software Foundation, https://www.python.org/downloads/) were used for the development of QAEmap.

## Supplementary Information

Supplementary Information.

## Data Availability

The coordinate and structure factor files can be downloaded from the Protein Data Bank (Supplementary Table S1). Output files from QAEmap for the simulated and experimental data that support the findings of this study are available from the corresponding author upon request.

## Code availability

The QAEmap program is freely available for academic use through GitLab (https://gitlab.com/qaemap_products/qaemap).
