# Supplementary material for: Machine learning to estimate the local quality of protein crystal structures
Source: Sci Rep. 2021 Dec 8;11:23599. doi: 10.1038/s41598-021-02948-y (PMC8654820; doi:10.1038/s41598-021-02948-y)
Supplement: Supplementary file 1 — Supplementary Information. [file 41598_2021_2948_MOESM1_ESM.pdf]

Supplementary Materials

Supplementary Table 1.

Protein structures and their homologous structures used for (a) training and (b) test data

(a) Data for training

PDB CODE	Reported resolution (Å)	Target chain	Number of amino acids	CATH code	Template pdbs (chain) for homology modeling						
1GQ1	1.4	A	559	1.10.760.10	1HZU(A)	1NIR(A)					
1Y0P	1.5	A	568	1.10.1130.10	1D4C(A)	1D4D(A)					
2BKL	1.5	A	676	3.40.50.1820	1E5T(A)	2XE4(A)	5N4B(A)	5O3U(A)			
2CAK	1.27	A	154	2.60.40.420	1A3Z(A)	1A8Z(A)	1GY1(A)				
2HOX	1.4	A	425	2.10.25.30	3BWN(A)						
2NLR	1.2	A	222	2.60.120.180	1H0B(A)	1NLR(A)	1OA4(A)	3WX5(A)			
2O6S	1.5	A	208	3.80.10.10	2V70(A)	3RFJ(A)	3RFS(A)	4J4L(A)	5B4P(A)		
2PPO	1.29	A	107	3.10.50.40	1BKF(A)	1BL4(A)	1C9H(A)	1FKB(A)	1FKK(A)	1KT1(A)	1N1A(A)
					1YAT(A)	3UQB(A)	3UQI(A)	5B8I(C)	5HW8(A)	5I7Q(A)	
2XPW	1.44	A	207	1.10.10.60	2TCT(A)	2VPR(A)					
2YNY	1.35	C	105	1.20.5.170	2WPQ(A)	3ZMF(A)					
3HIS	1.49	A	257	3.40.420.10	1APA(A)	1LP8(A)	1PAF(A)	1QI7(A)	2Q8W(A)	3H5K(A)	3HIQ(A)
3LKY	1.11	A	123	2.100.10.30	2GUX(A)	2HYQ(A)	3LL1(A)	3LL2(A)			
3OG2	1.2	A	986	3.20.20.80							
3T9G	1.5	A	196	2.160.20.10	4YZ0(A)	4Z03(A)					
3VL9	1.2	B	221	2.60.120.180	1H8V(A)	1KS4(A)	1OA2(A)	1OA3(A)	1OLR(A)	4NPR(A)	5GM3(A)
					5GM4(A)	5M2D(A)					
3WW9	1.33	A	251	2.120.10.30	1RWI(B)	6F0S					
3ZTP	1.37	A	141	3.30.70.141	1B4S(A)	1BE4(A)	1JXV(A)	1K44(A)	1LEO(A)	1NCL(A)	1PKU(A)
					1S57(A)	1U8W(A)	2NCK(R)	3EMT(A)	3PJ9(A)	3VGS(A)	4S0M(A)
4AC7	1.5	B	122	3.30.280.10	1A5K(B)	2KAU(B)	4G7E(A)	4Z42(B)			
4TR6	1.5	A	380	3.10.150.10	1JQL(A)	2AVT(A)	2AWA(A)	3T0P(A)	4TR8(A)	4TRT(A)	5W7Z(A)
					5WCE(A)						
5CEC	1.36	A	401	3.40.710.10	5CEB(A)	5CER(A)					
5D7W	1.1	A	469	2.150.10.10	1AF0(A)	1GO7(A)	1JIW(P)	1K7G(A)	1SAT(A)		
5M1P	1.1	B	189	3.30.420.240	5TGE(A)						

(b) Data for test

PDB CODE	Reported resolution (Å)	Target chain	Number of amino acids	CATH code	Template pdbs (chain) for homology modeling				
1LC0	1.2	A	294	3.40.50.720	1LC0(A)	2H63(A)			
3F9X	1.25	D	166	2.170.270.10	1ZKK(A)	3F9X(D)	3F9Z(A)	4LDG(A)	5V2N(A)
1D5T	1.04	A	433	3.50.50.60	6C87(A)				
3BUX	1.35	B	305	1.20.930.20<br>1.10.238.10<br>3.30.505.10	6BK5(A)				
4RDJ	1.5	B	297	2.40.510.10	5OR7(A)				
5KWN	1.5	A	326	2.130.10.10	5IGQ(A)				
5MFI	1.45	B	240	1.25.10.10	4RZP(A)				

Incorrect model struct.
(1.25 Å reso.)

Incorrect model struct.
(3.0 Å reso.)

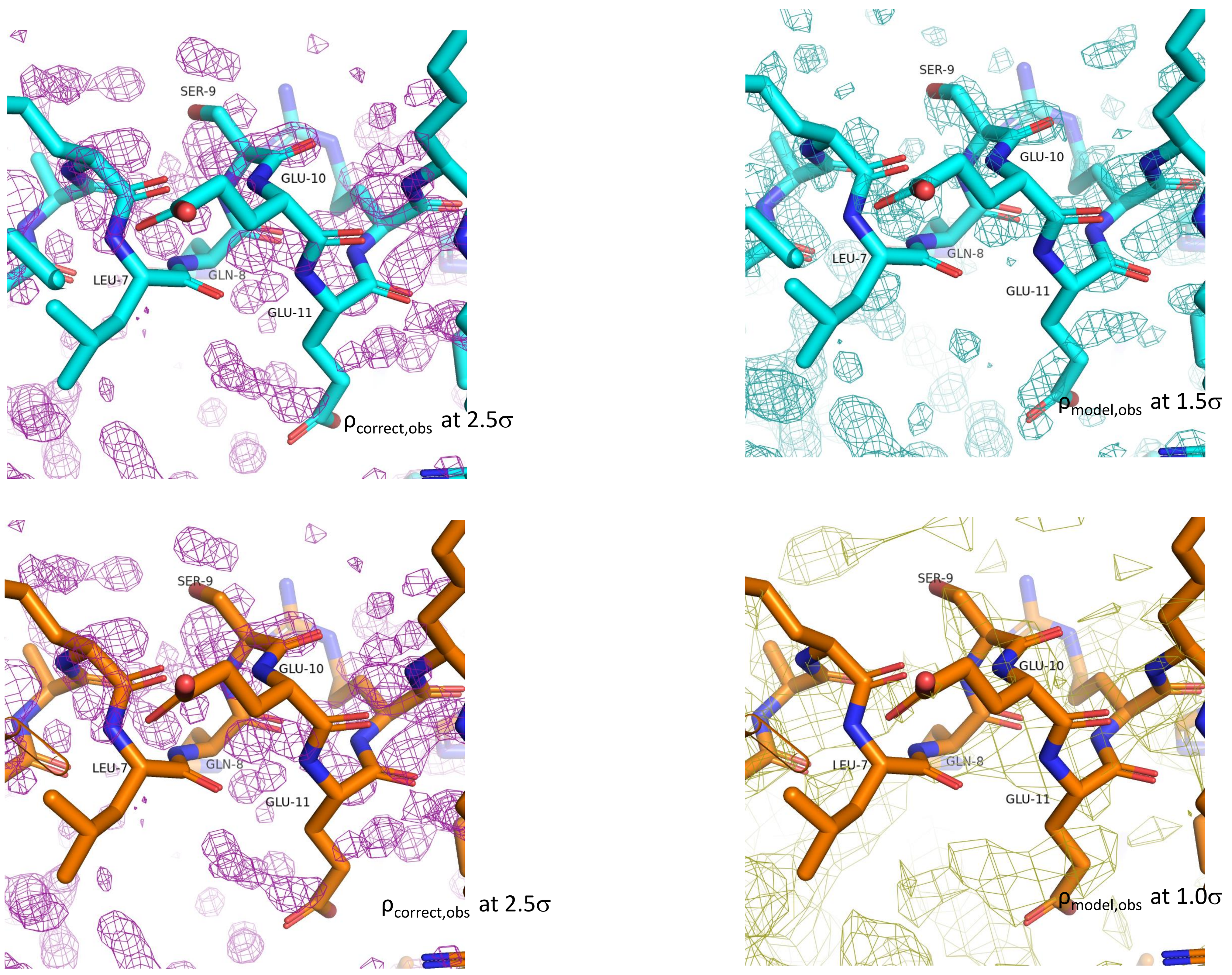

Supplementary Fig. 1

Electron density maps and coordinates for the calculation of bCC and RSCC in Fig. 3b. On the left, the correct electron density and the incorrect model structure at each resolution; on the right, the electron density of each model structure. The low-resolution map is more affected by the model bias.

(a)

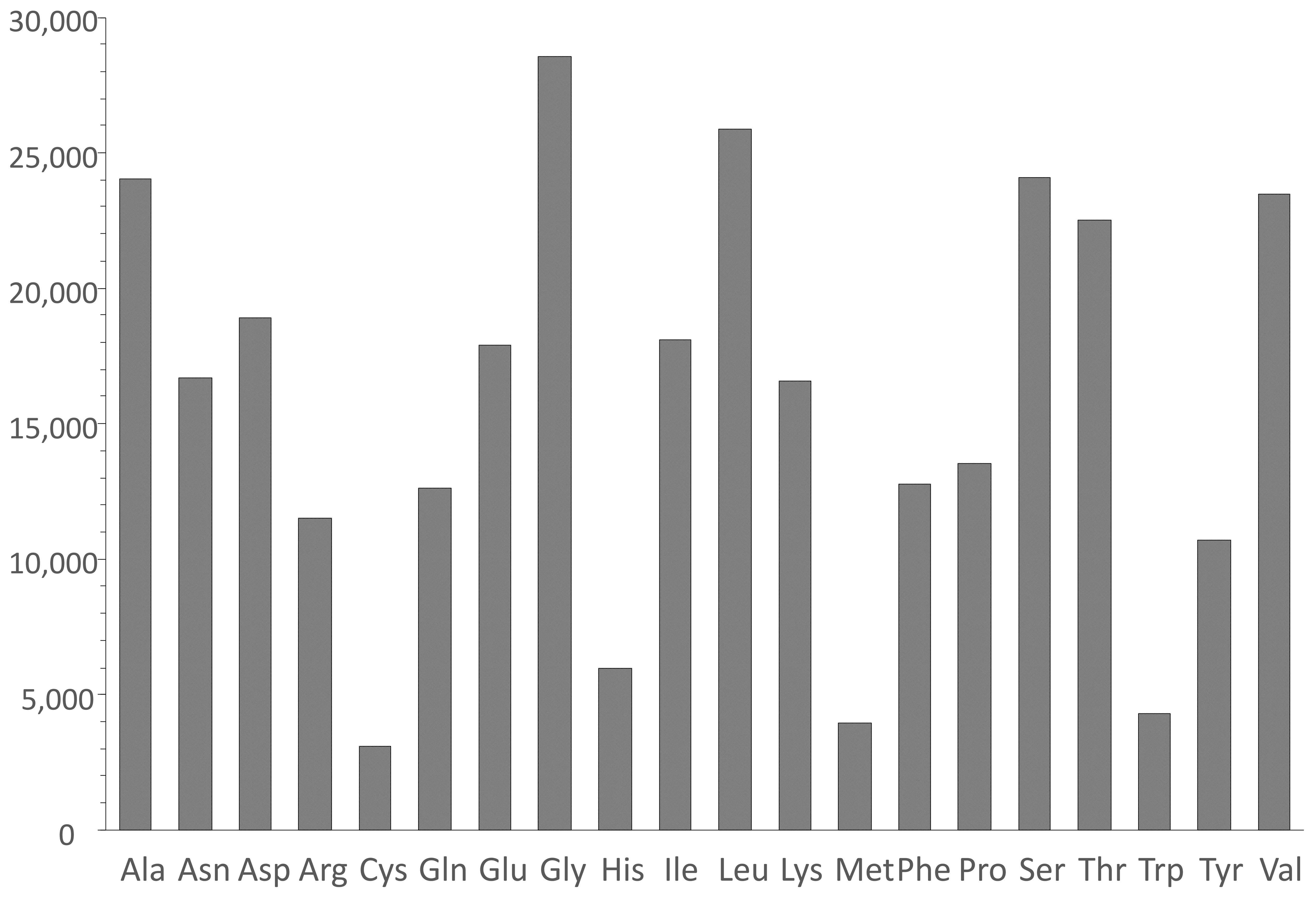

(b)

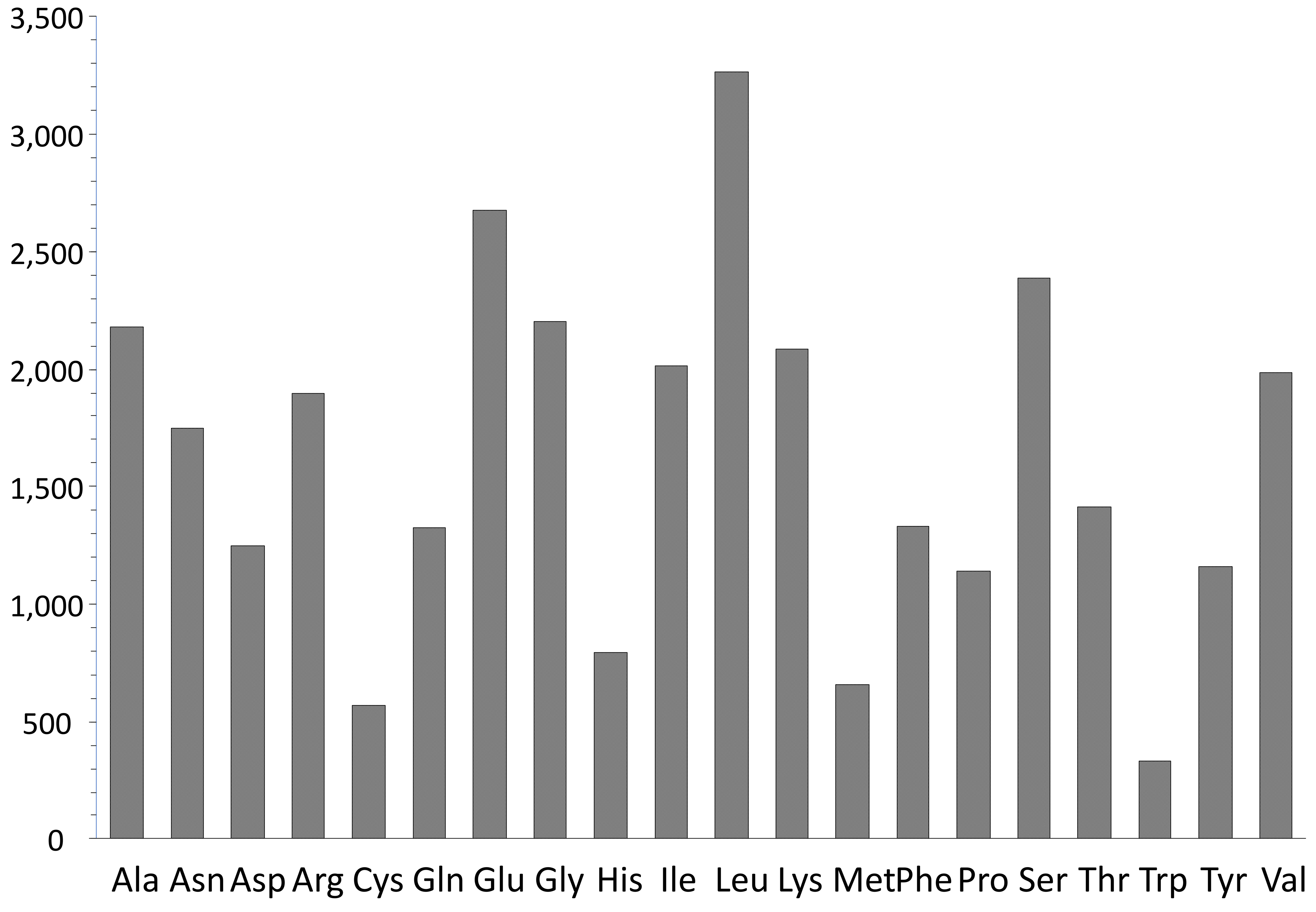

Supplementary Fig. 2

Distributions of the amino acids in **a** (training data) and **b** (test data)

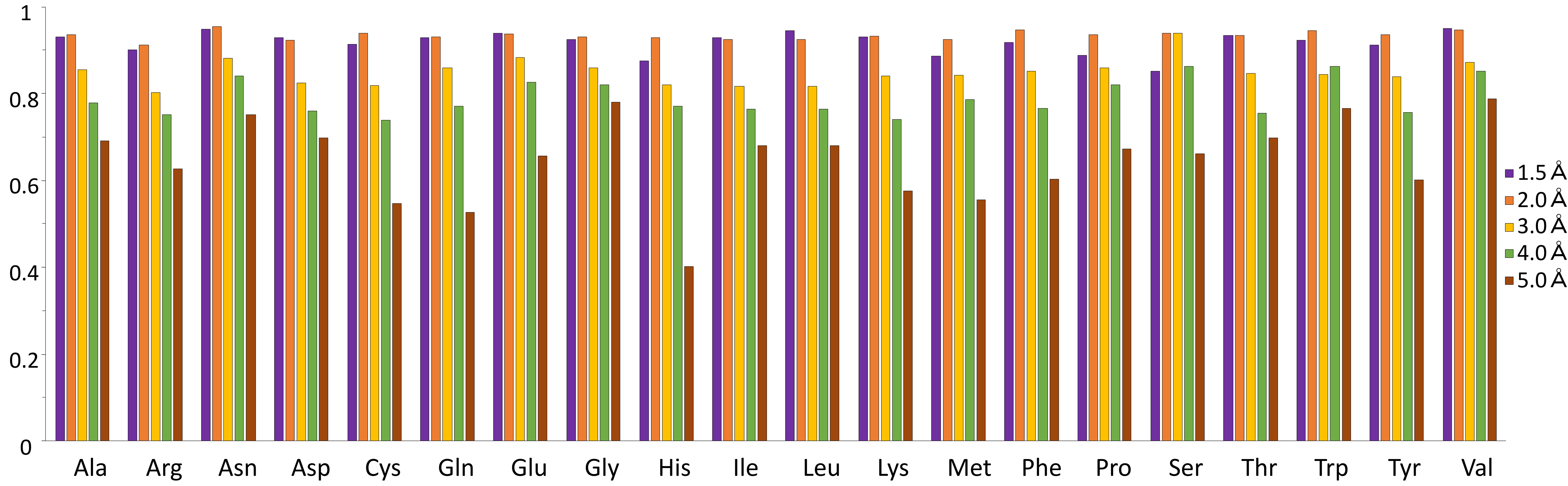

Supplementary Fig. 3

Correlation coefficients between $bCC_{act.}$ and $bCC_{pred.}$ based on amino acid and resolution

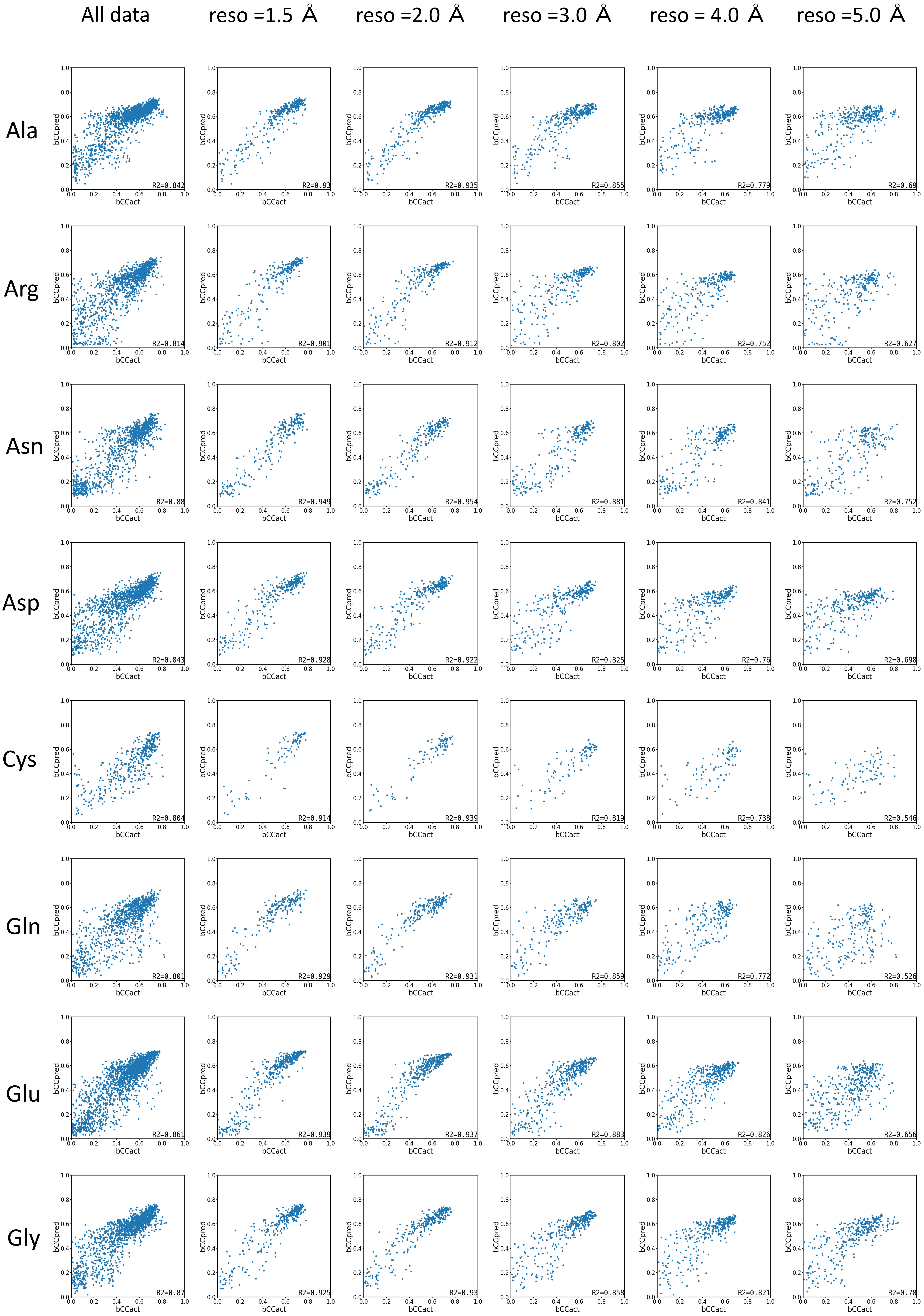

Supplementary Fig. 4

Correlation between $bCC_{act.}$ (horizontal axis) and $bCC_{pred.}$ (vertical axis) based on the amino acids and resolution.

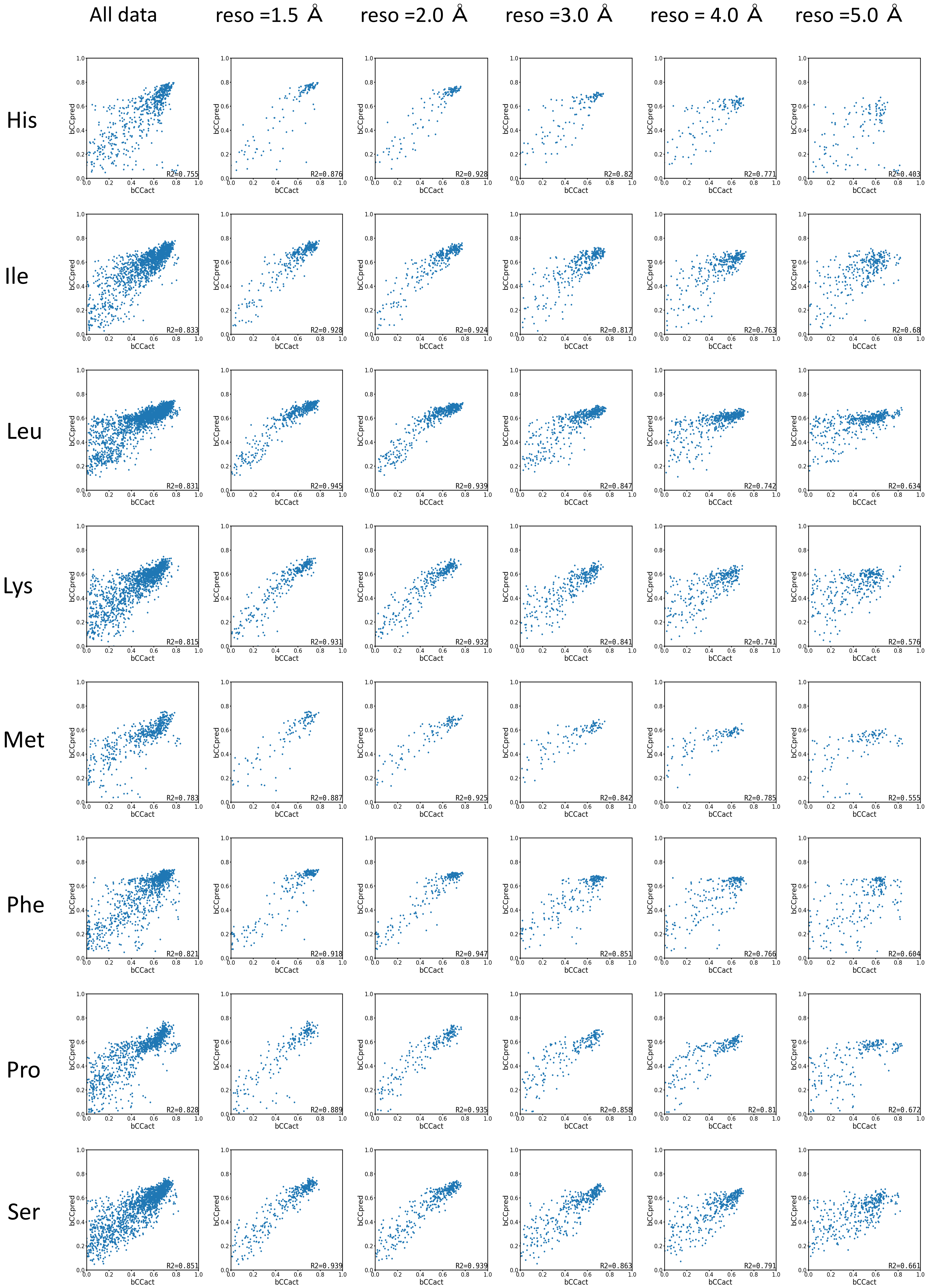

Supplementary Fig. 4 (Continued)

Correlation between $bCC_{act.}$ (horizontal axis) and $bCC_{pred.}$ (vertical axis) based on the amino acids and resolution.

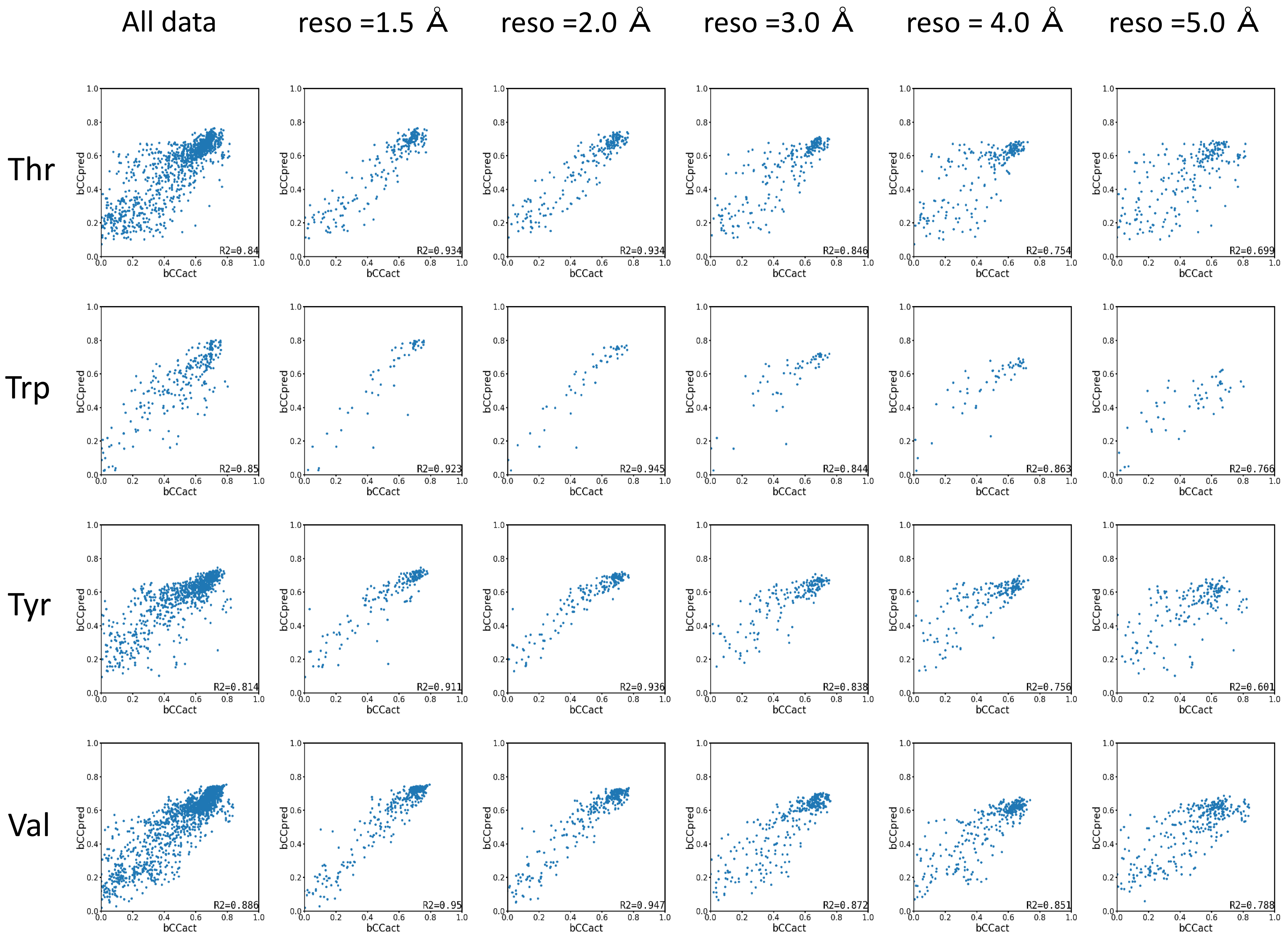

Supplementary Fig. 4 (Continued)

Correlation between $bCC_{act.}$ (horizontal axis) and $bCC_{pred.}$ (vertical axis) based on the amino acids and resolution.

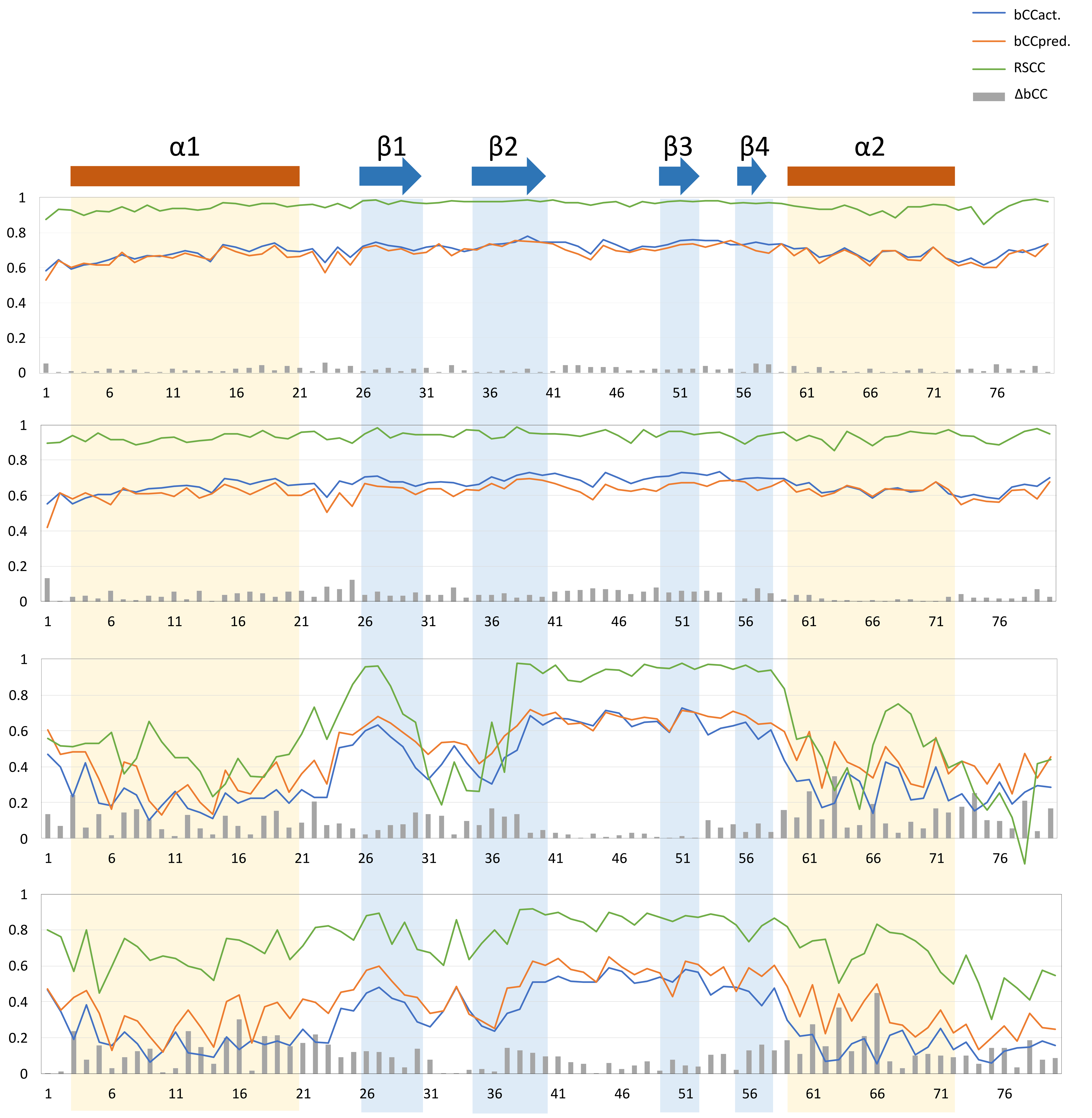

Supplementary Fig. 5

Values of $bCC_{pred.}$, $bCC_{act.}$, and RSCC in the four test structures. Differences between $bCC_{pred.}$ and $bCC_{act.}$ are also shown. From the top: 3F9X_correct(1.25 Å resolution), simulated low resolution structure(3.0 Å), model structures, which refined at 1.25 Å and 3.0 Å resolution of structure factors.

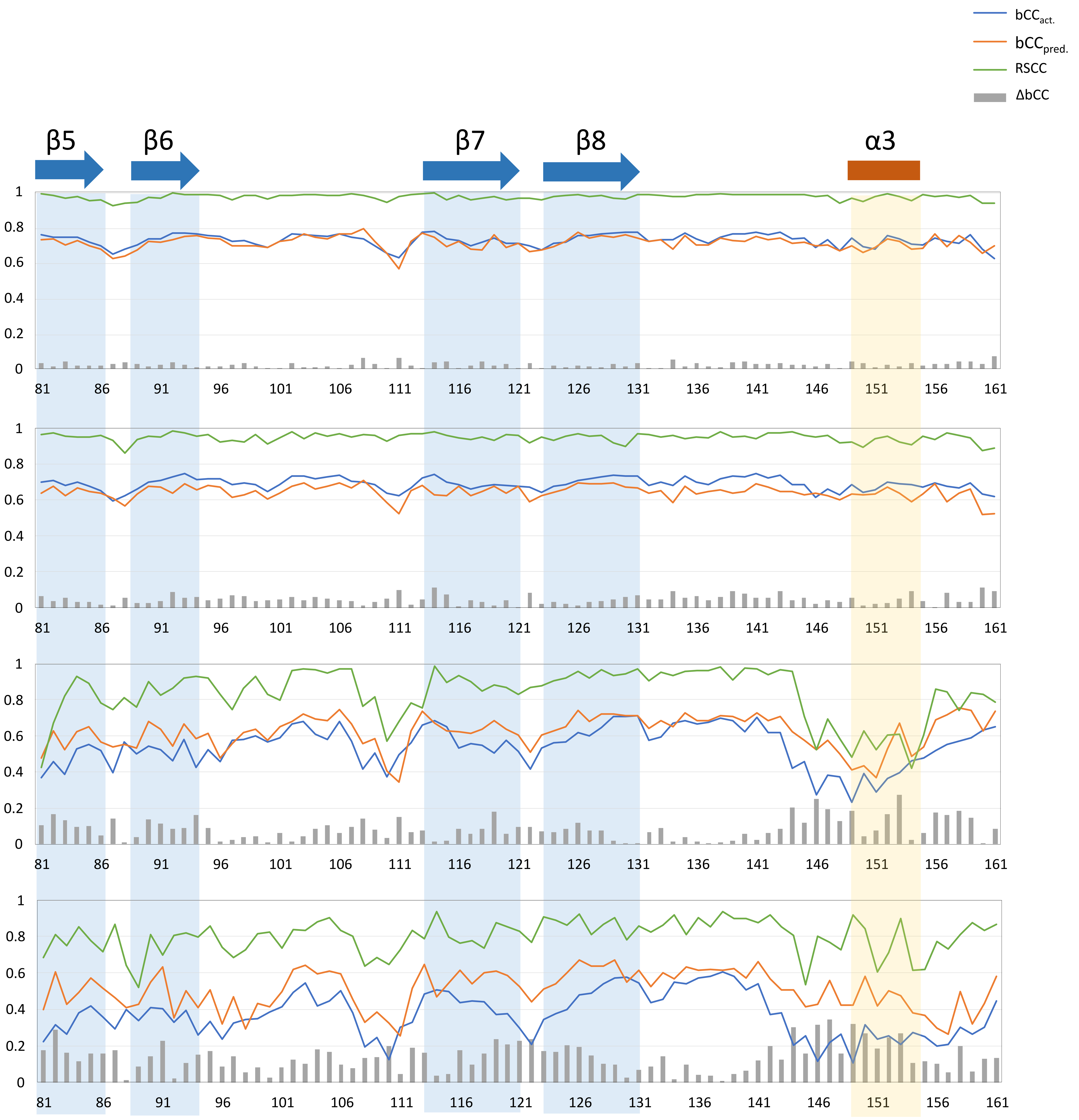

Supplementary Fig. 5 (Continued)

Values of $bCC_{pred.}$, $bCC_{act.}$, and RSCC in the four test structures. Differences between $bCC_{pred.}$ and $bCC_{act.}$ are also shown. From the top: 3F9X_correct(1.25 Å resolution), simulated low resolution structure(3.0 Å), model structures, which refined at 1.25 Å and 3.0 Å resolution of structure factors.

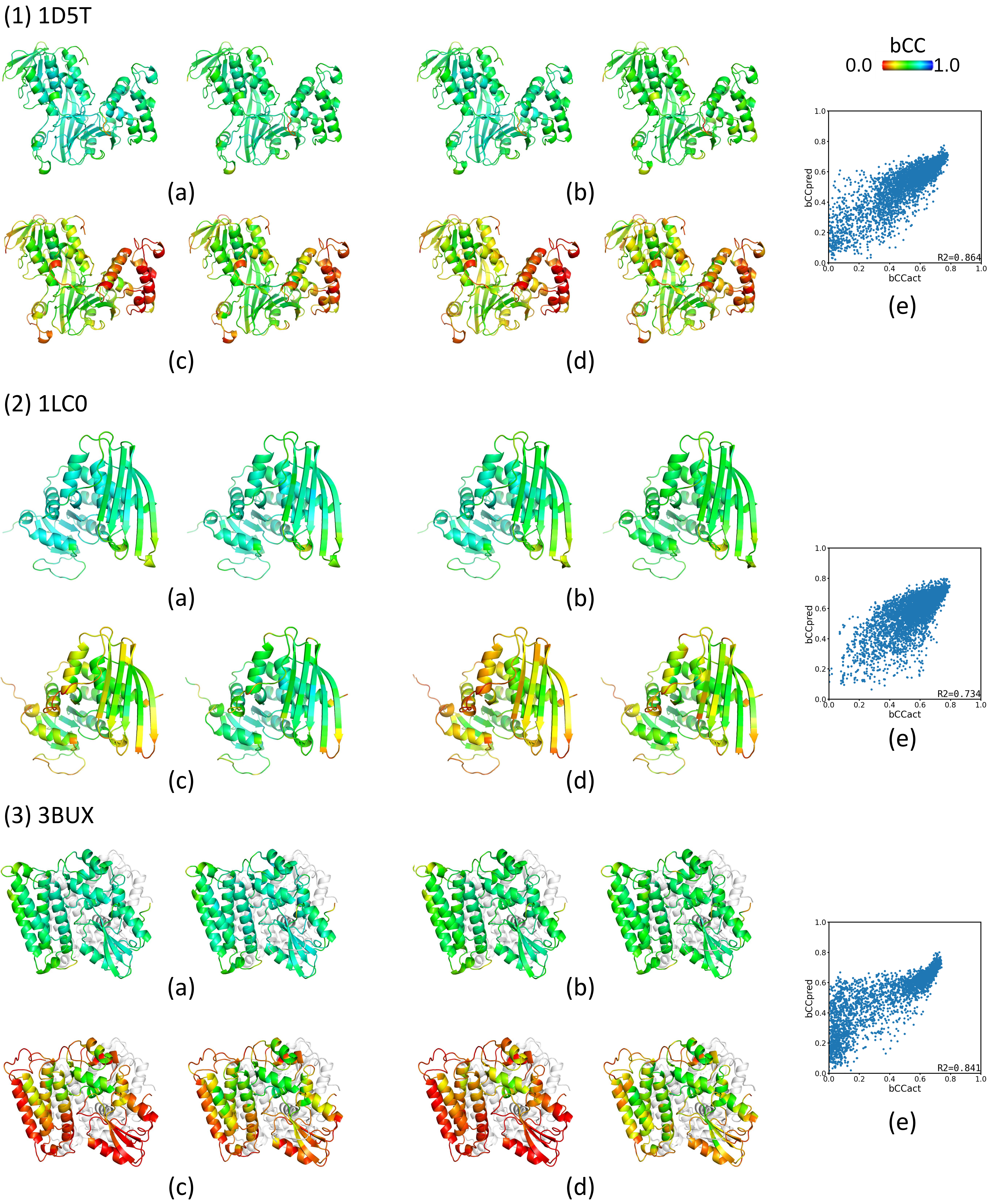

Supplementary Fig. 6

Evaluation by QAEmap. In each pair of (a) – (d), the molecule on the left is colored with bCC_{act}, and the left is with $bCC_{pred.}$ (a) 1.5-Å correct structure, (b) 3.0-Å correct structure, (c) 1.5-Å incorrect model structure, (d) 3.0-Å incorrect model structure, (e) Correlation between bCC_{act}. and $bCC_{pred.}$ of all data at resolution 1.5, 2.0, 3.0, 4.0 and 5.0 Å.

(4) 4RDJ

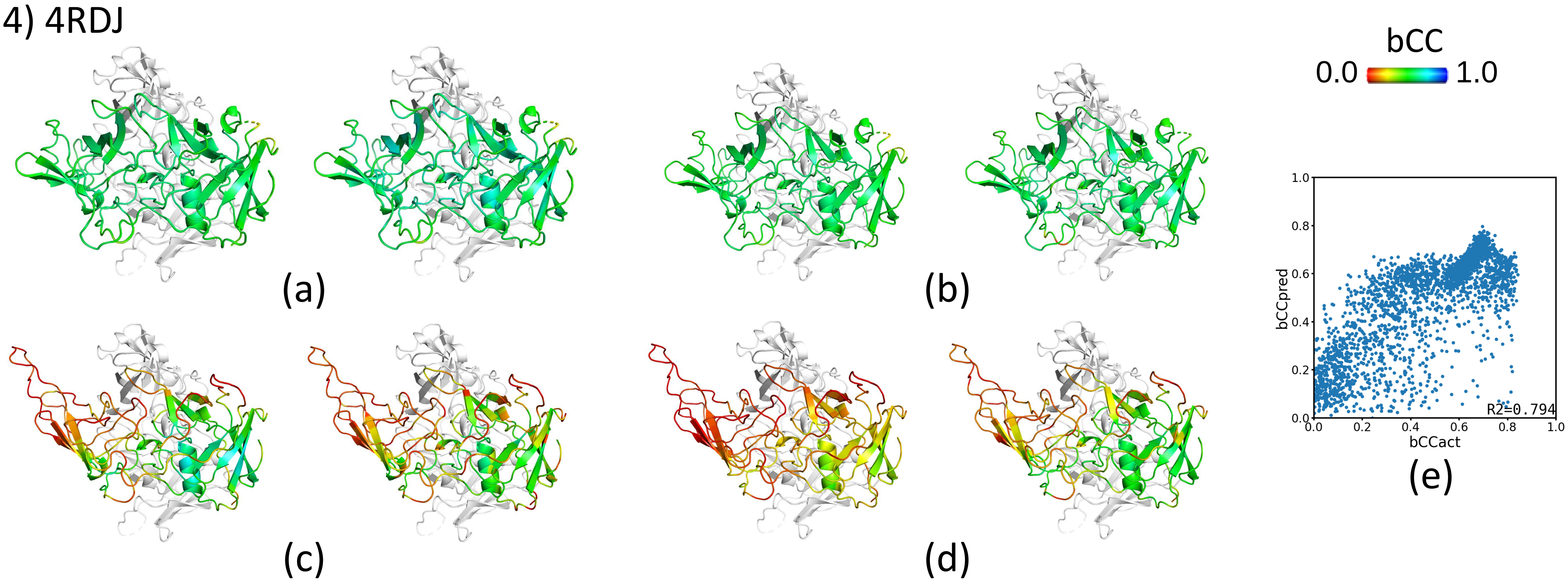

(5) 5KWN

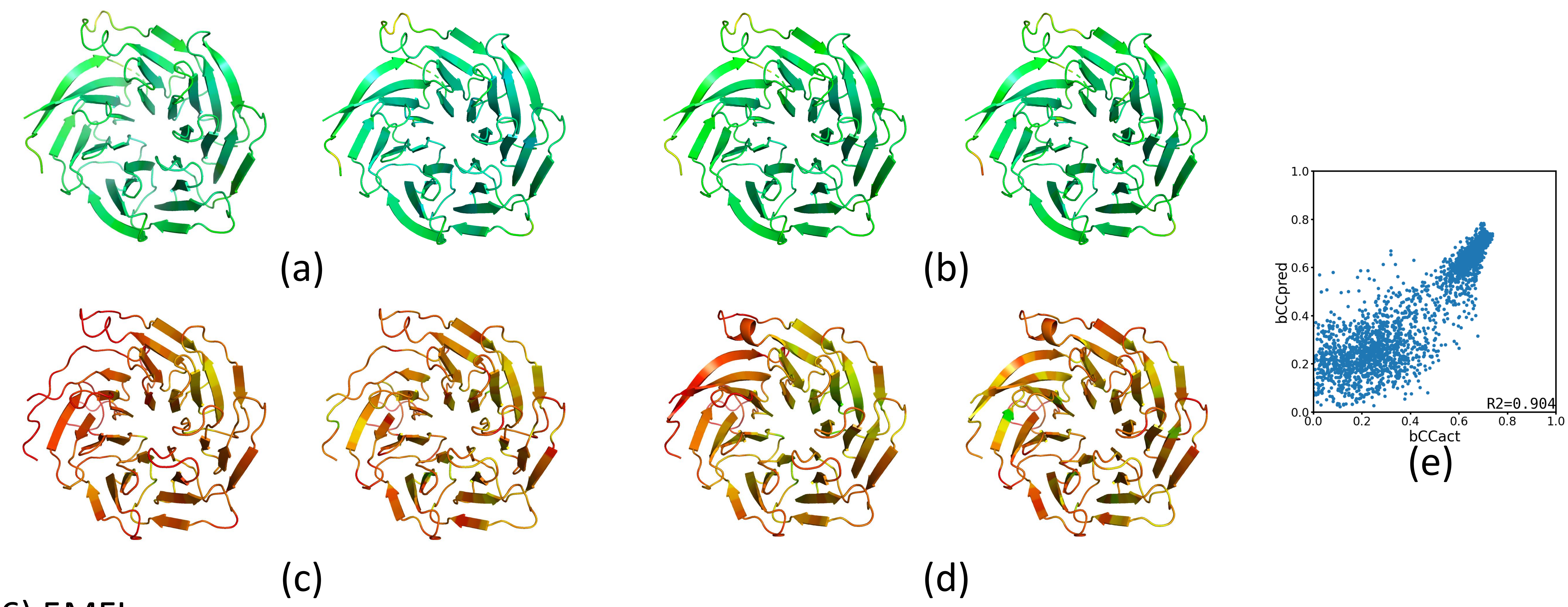

(6) 5MFI

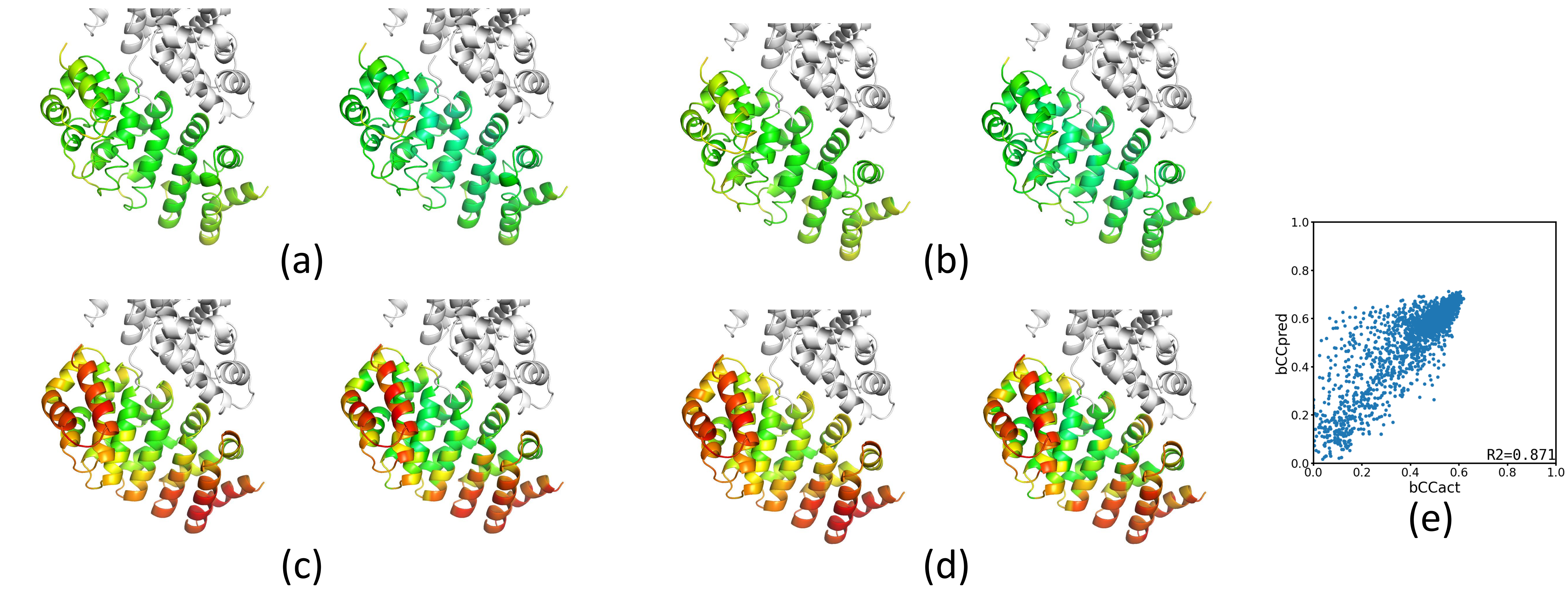

Supplementary Fig. 6 (Continued)

Evaluation by QAEmap. In each pair of (a) – (d), the molecule on the left is colored with bCC_{act}, and the left is with $bCC_{pred.}$ (a) 1.5- Å correct structure, (b) 3.0- Å correct structure, (c) 1.5- Å incorrect model structure, (d) 3.0- Å incorrect model structure, (e) Correlation between $bCC_{act.}$ and $bCC_{pred.}$ of all data at resolution 1.5, 2.0, 3.0, 4.0 and 5.0 Å.

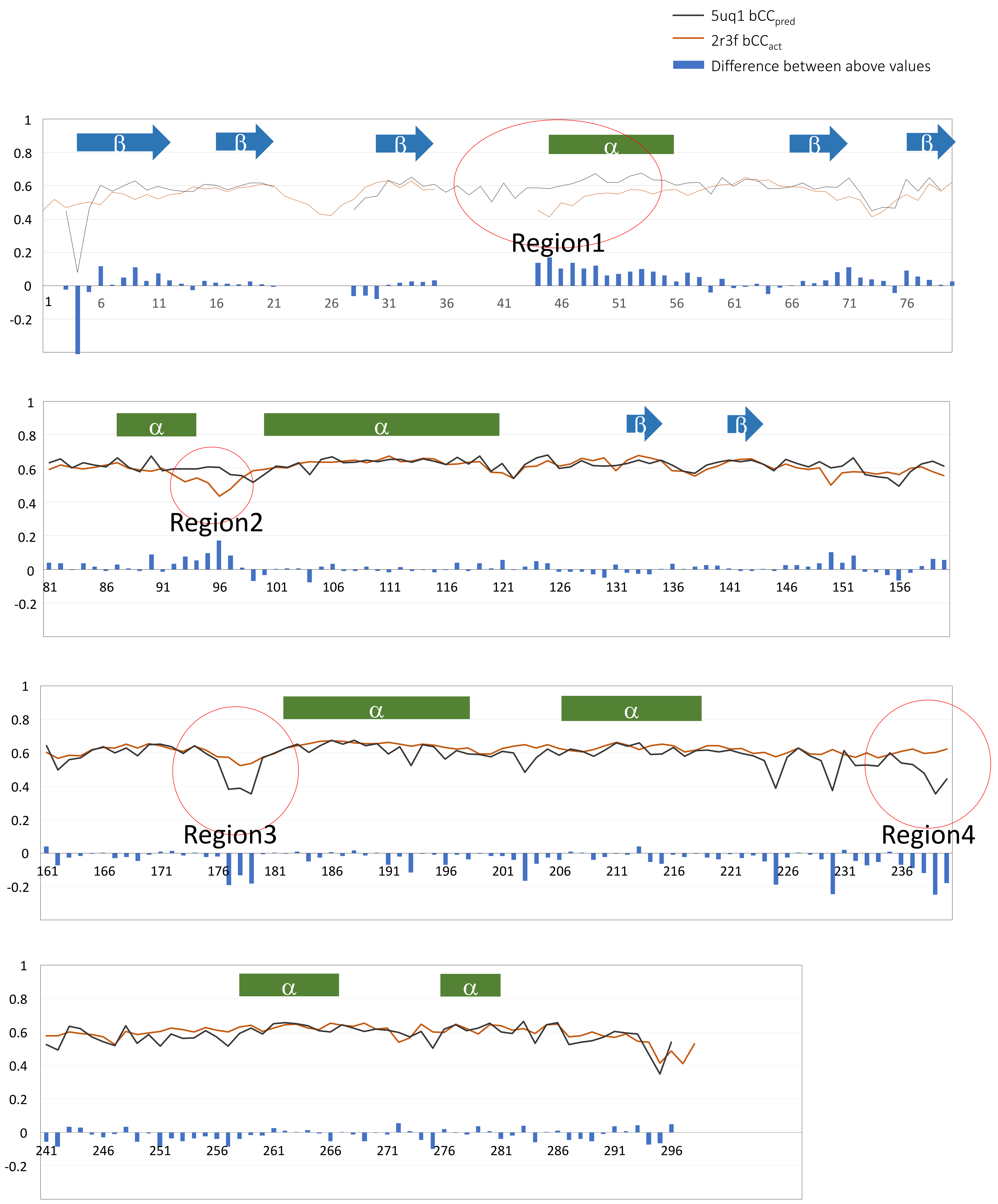

Supplementary Fig. 7

Comparison of two CDK2 structures. $bCC_{pred.}$ of 5UQ1(3.2 Å resolution) and $bCC_{act.}$ of 2R3F(1.5 Å resolution) are shown. Region 3 is discussed in Fig. 6c in the text, and regions 1, 2 and 4 are discussed in the text as well as in Supplementary Fig. 8.

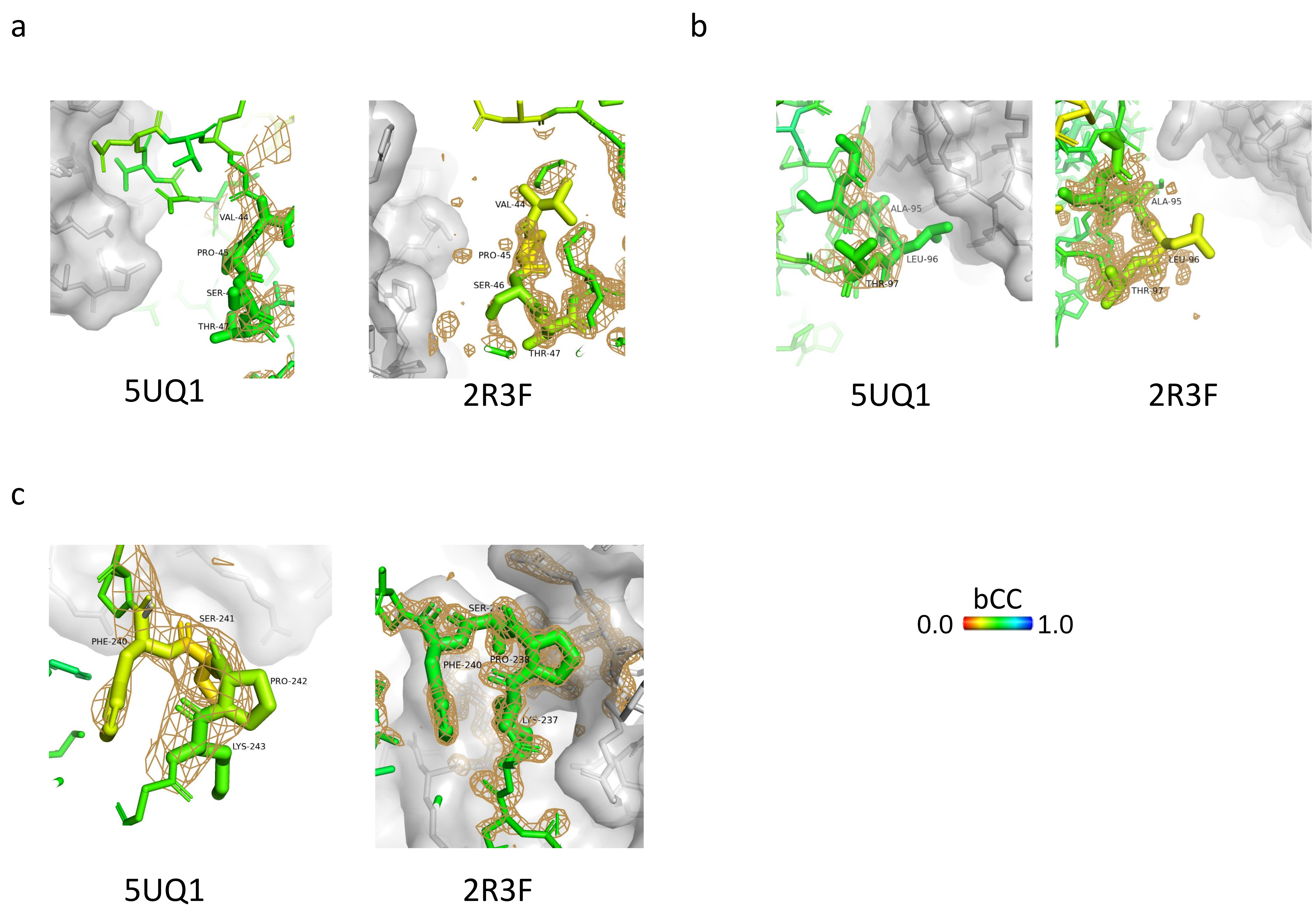

Supplementary Fig. 8

Amino acids evaluated by bCC (in sticks colored by $bCC_{pred.}$ for 5UQ1 and $bCC_{act.}$ for 2R3F) and their electron density, along with their neighboring molecule in the crystal (in gray surface presentation) **a** (Region1), **b** (Region 2), **c** (Region 4) in Supplementary Fig. 7. bCC is high when the amino acids interact with the neighboring molecules and low when they are exposed to the solvents.

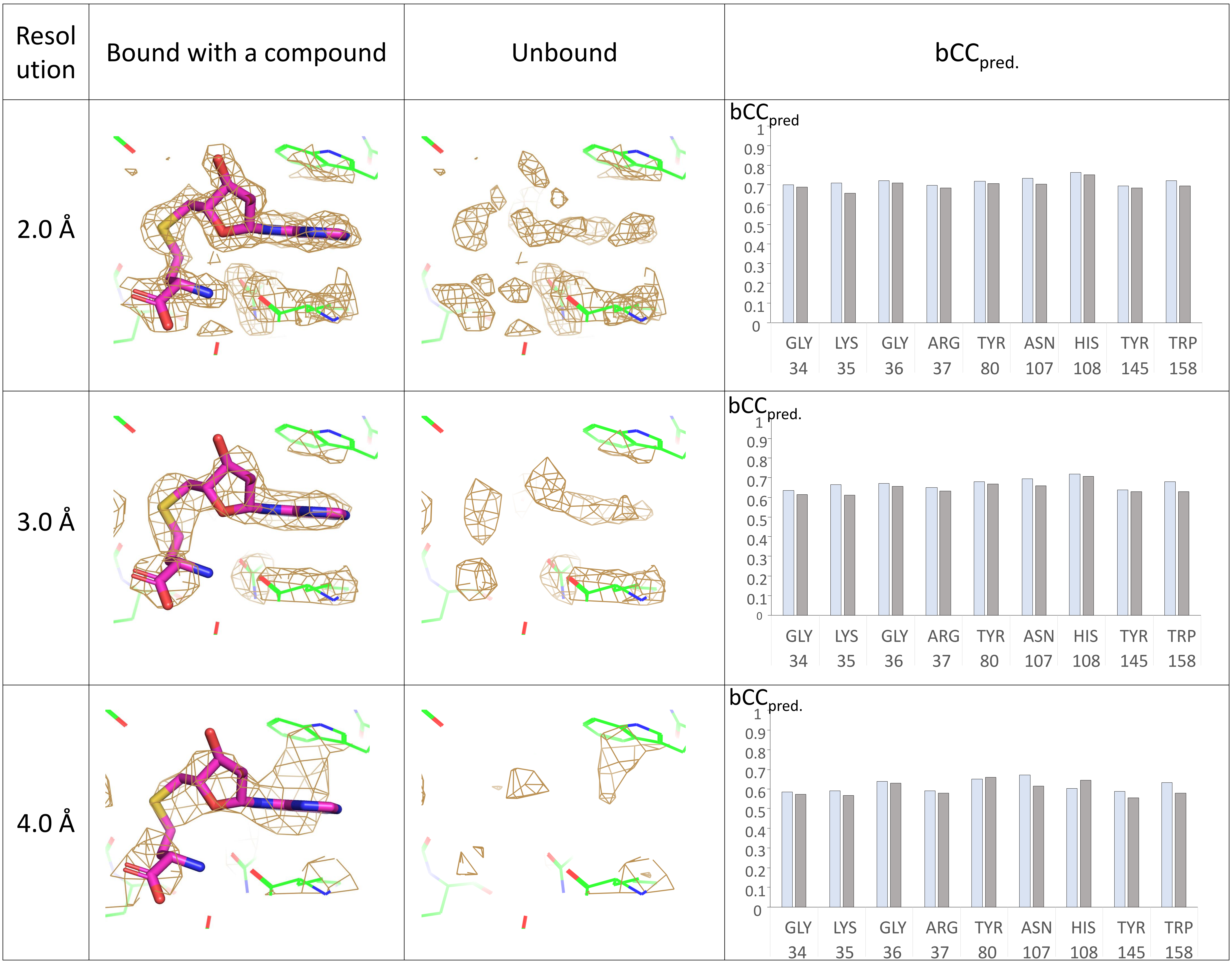

Supplementary Fig. 9

Coordinates and σ_{obs} electron densities for a SAH molecule at 1.5σ and $bCC_{pred.}$ values for the nine residues with and without SAH in the model at 2.0, 3.0 and 4.0 Å resolutions.
